# Effects of Marathon Running on Skin and Plasma Carotenoids in Endurance Runners

**DOI:** 10.3390/nu18030437

**Published:** 2026-01-29

**Authors:** Damon Joyner, Tracy M. Covey, Leigh Komperda, Margarita Lopez, Saori Hanaki, Bryan Dowdell, Stacie Wing-Gaia, Qi Jin, Jamie Stein, David Aguilar

**Affiliations:** 1 Department of Exercise and Nutrition Sciences, Weber State University, 1435 Village Dr., Ogden, UT 84403, USA; damonjoyner@weber.edu (D.J.); margaritalopez@weber.edu (M.L.); saorihanaki@weber.edu (S.H.); bryandowdell@weber.edu (B.D.); swinggaia@weber.edu (S.W.-G.); qijin1@weber.edu (Q.J.); jamiestein@weber.edu (J.S.); 2 Department of Chemistry and Biochemistry, Weber State University, 1415 Edvalson St., Ogden, UT 84403, USA; tracycovey@weber.edu (T.M.C.); leighkomperda@weber.edu (L.K.)

**Keywords:** carotenoids, beta-carotene, lycopene, marathon, exercise, phytochemicals, oxidative stress, runners

## Abstract

Background/Objectives: Carotenoids are pigmented phytochemicals known for their antioxidant properties, known to protect against oxidative damage, especially in the context of intense exercise. The purpose of this paper was to observe and analyze the short-term effects of running a full marathon on skin and plasma carotenoid levels in endurance runners. Methods: This study recruited 24 healthy endurance runners (12 male, 12 female; mean age 37 years) registered for a 26.2-mile marathon. Skin carotenoid (SC) measures were taken via reflection spectroscopy, and plasma carotenoid concentrations (lycopene and β-carotene) were assessed via HPLC at three time points: pre-race, immediately post-race, and 48 h post-race. Changes across time were analyzed using repeated-measures ANOVA. Results: Skin carotenoid levels significantly changed over time, dropping from pre-race to post-race (*p* < 0.001), reflecting rapid utilization. At 48 h post-race, SC levels rebounded significantly, exceeding baseline measurements (*p* = 0.019). Plasma lycopene concentrations increased significantly from pre-race to post-race (*p* = 0.018) and remained elevated at 48 h. Plasma β-carotene concentrations showed no statistically significant change. Conclusions: The significant acute depletion of SC levels immediately following the marathon reflects the rapid utilization of these dermal antioxidants in response to the high oxidative stress generated by intense exercise. The elevation in plasma lycopene may reflect hemoconcentration resulting from intense activity and possible mobilization from tissue stores. The rapid rebound and overshoot in SC levels 48 h after the race are consistent with a recovery pattern of dermal carotenoid levels following acute depletion.

## 1. Introduction

Carotenoids, a diverse group of pigmented phytochemicals, are widely recognized for their antioxidant properties and potential health benefits. These compounds, which include β-carotene, lutein, lycopene, and others, play a vital role in protecting cells from oxidative damage by neutralizing reactive oxygen species (ROS) [1,2]. In addition to antioxidant activity, some carotenoids, particularly β-carotene, function as provitamin A compounds and contribute to vitamin A status through enzymatic conversion in human tissues [3]. Moreover, carotenoids have been shown to contribute to overall health by influencing immune function, reducing inflammation, and protecting against various chronic diseases such as cardiovascular disease, cancer, and neurodegeneration [4]. The benefits of carotenoids are particularly relevant in the context of exercise, where increased metabolic activity leads to the production of ROS and other oxidative stress markers that can damage muscle tissue and impair performance [5].

Exercise-induced oxidative stress has been a significant area of interest in sports nutrition and exercise physiology, as it can both challenge the body’s antioxidant defenses and contribute to muscle fatigue, inflammation, and injury [6]. Recent studies have explored the potential role of phytochemicals and carotenoids in mitigating oxidative stress induced by physical exertion, suggesting that carotenoid supplementation may enhance recovery, reduce muscle damage, and improve overall performance [7,8,9]. These findings raise important questions regarding the efficacy of carotenoids in the diet for athletes and physically active individuals, particularly in terms of optimizing exercise outcomes and preventing long-term damage from oxidative stress.

While the mechanisms by which carotenoids exert their effects are still being explored, the potential for carotenoid-rich foods to improve exercise performance and recovery continues to gain attention. The purpose of this paper is to further the relationship between carotenoids and endurance exercise by observing and analyzing the effects that a full marathon has on skin and plasma carotenoid levels in marathon runners. We hypothesized that skin carotenoid scores would decrease immediately post-marathon and rebound during early recovery (48 h), while plasma carotenoids would show minimal change or modest increases immediately post-marathon.

## 2. Materials and Methods

### 2.1. Participants

Male and female participants ages 18–65 years were recruited from the GOAL Foundation (Ogden, UT, USA), the organization responsible for hosting the annual Ogden Marathon. Twenty-four healthy participants were recruited to participate in this study, none of which had underlying disease or deleterious conditions. Sex was split equally (12 male, 12 female), with a mean age of 37 (±10) years. All participants were active, with the majority (74%) considering themselves trained or highly trained athletes. See Table 1 for participant demographics.

### 2.2. Study Procedure

**Figure 1:**
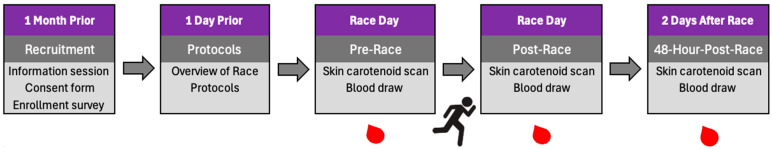
Research Timeline.

Each participant was registered for and ran the 26.2-mile Ogden Marathon. Each participant was required to complete skin and plasma carotenoid measures during three timeframes relative to the marathon race: pre-race (three hours prior), post-race (immediately after finishing the marathon), and 48 h post-race (two days after completing the marathon). See Figure 1 for the research timeline.

Skin carotenoid assessment and venous blood collection were performed within approximately 10–15 min of marathon completion using a consistent order across participants. Participants were allowed to consume water ad libitum during and immediately after the race; fluid intake and acute supplement use were not standardized. Marathon finish time ranged from approximately 170 to 400 min.

Body mass and total body water were measured using bioelectrical impedance analysis (InBody) immediately before the race, immediately post-race, and 48 h post-race to characterize hydration-related changes associated with marathon participation.

### 2.3. Carotenoid Measures

Skin carotenoids were measured via reflection spectroscopy (RS). RS is a non-invasive, validated method for assessing stored amounts of carotenoids in the skin, which are a biomarker for fruit and vegetable (FV) intake and are highly correlated with plasma high-performance liquid chromatography carotenoid levels [10,11,12,13]. Carotenoid levels are reported on a scale from 0 to 800, with higher numbers indicating greater carotenoid storage in the skin. Measurements were obtained pre-race, immediately post-race, and 48 h post-race. Skin carotenoids were assessed using the Veggie Meter^®^ (Longevity Link Corporation, Salt Lake City, UT, USA), which employs pressure-mediated reflection spectroscopy. The instrument was calibrated according to the manufacturer’s procedures using the supplied reference standard prior to data collection. Measurements were collected on the index finger of the same hand at all time points, with three consecutive readings averaged to derive a single skin carotenoid score per time point, following standardized protocols described in prior validation studies [12,14].

Each participant also had a pre-race, post-race, and 48 h post-race blood draw to assess plasma carotenoid levels and blood was stored at −80 °C prior to analysis. The human plasma carotenoid (β-carotene, lycopene) analysis was performed by HPLC. Plasma carotenoids were extracted using β-carotene in Serum/Plasma extraction kit (#32000) (Chromsystems Instruments & Chemicals GmbH, Grafelfing, Germany) following the manufacturer’s protocols. Briefly, 100 µL of calibrator, control, or sample was mixed with 50 µL internal standard and mixed briefly. A total of 50 µL of precipitation reagent was added and mixed, followed by 200 µL of extraction buffer. The samples were spun at 16,000× *g* for 10 min. The supernatants were transferred to an HPLC vial for analysis. A total of 5 µL of supernatant was used for injection volumes on UHPLC.

Carotenoids were analyzed by UHPLC using a Thermo Vanquish UHPLC (Thermo Fisher Scientific, Bremen, Germany), comprising a quaternary pump, an online degasser, a column oven controller, and a diode-array detector (DAD). Carotenoids were separated on a reverse-phase C18, 1.8 μm column (100 × 1.0 mm) (Waters Corporation, Milford, MA, USA) using an isocratic mobile phase consisting of β-Carotene Mobile Phase (#32001-C) (Chromsystems Instruments & Chemicals GmbH, Grafelfing, Germany) at a flow rate of 0.2 mL/min. The column temperature was maintained at 25 °C. The eluting peaks were monitored at 453 nm using DAD. Quantification was performed using Chromeleon software version 7.2.10 ES from Thermo Fisher Scientific, Bremen, Germany., comparing peak area with internal standard and calibration curves.

Dietary carotenoid intake was assessed using multiple 24 h dietary recalls collected at four time points: two days prior to the race, the day before the race, the day of the race, and 48 h post-race. Dietary and supplement data were collected using the Automated Self-Administered 24 h Dietary Assessment Tool (ASA24), which has been validated for dietary tracking [15]. The ASA24 system was used to estimate daily intakes of β-carotene and lycopene based on reported food and beverage consumption. All recalls were reviewed for completeness and plausibility before analysis. Average carotenoid intake across time points was calculated and used for comparison with plasma and skin carotenoid measures. Analyses were performed with the Python programming language version 3.11.13 using statistical and graphics packages. The libraries Scikit-learn version: 1.6.1, Numpy version: 2.0.2, and Pandas version: 2.2.2 were used for the analysis of the data.

### 2.4. Statistical Analysis

A repeated-measures one-way ANOVA was used to determine whether differences in carotenoid intake, skin, and plasma existed across measurement phases. Comparisons were performed using Least Significant Difference (LSD) post hoc tests, with *p* < 0.05 considered statistically significant. All analyses were made using SPSS 29 for Mac (Armonk, NY, USA: IBM Corp.).

## 3. Results

### 3.1. Skin Carotenoids

**Figure 2:**
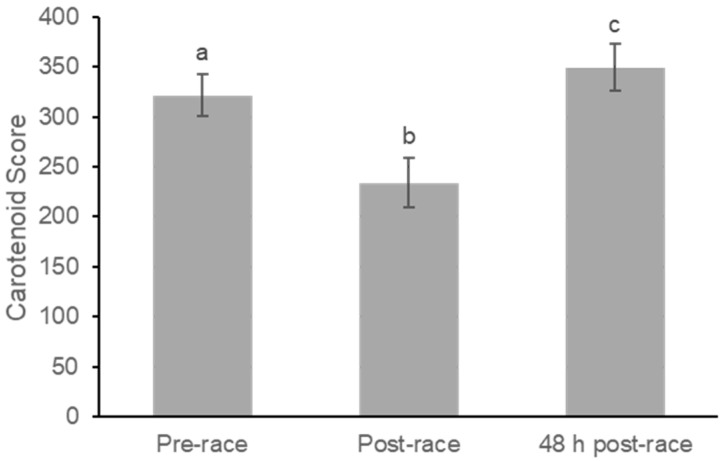
Changes in Skin Carotenoids Before, Immediately After, and 48 h Following Marathon Participation. Skin carotenoids were measured using reflection spectroscopy at pre-race, immediately post-race, and 48 h post-race. Values are mean ± SE. Skin carotenoids significantly decreased immediately after the marathon and increased at 48 h compared with both pre-race and post-race values. Bars with different letters indicate significant differences between time points (*p* < 0.05).

A repeated-measures one-way ANOVA was used to analyze changes in skin carotenoid (SC) levels across the three measurement phases (pre-race, post-race, and 48 h post-race). Skin carotenoid levels significantly changed over time (F(2,46) = 61.28, *p* < 0.001; partial η^2^ = 0.73) (Figure 2).

Baseline SC levels (pre-race) were scored at 320.125 (SE = 22.390), with a 95% confidence interval (CI) between 273.808 and 366.442. Immediately after the marathon (post-race), SC levels dropped sharply to a mean of 229.292 (SE = 25.455), with a 95% CI between 176.633 and 281.950. Pairwise comparisons using the LSD adjustment showed that the decrease from pre-race to post-race was highly significant (mean difference = 90.833, SE = 12.302, *p* < 0.001), representing a very large within-subject effect (Cohen’s dz = 1.51).

Forty-eight hours after the marathon (48 h post-race), SC levels rebounded significantly, reaching a mean score of 349.750 (SE = 23.045), with a 95% CI between 302.077 and 397.423. This value exceeded the baseline measurement, demonstrating a significant recovery and slight elevation relative to the pre-race level. The difference between the 48 h post-race level and the pre-race level was statistically significant (mean difference = 29.625, SE = 11.703, *p* = 0.019) and corresponded to a moderate effect size (dz = 0.52). Additionally, the increase from post-race to 48 h post-race was highly significant (mean difference = 120.458, SE = 9.872, *p* < 0.001), reflecting an extremely large effect (dz = 2.49).

Overall, the pattern observed for skin carotenoids was an acute decrease after the marathon, followed by an overshoot recovery above baseline values.

### 3.2. Plasma Carotenoids: Lycopene

**Figure 3:**
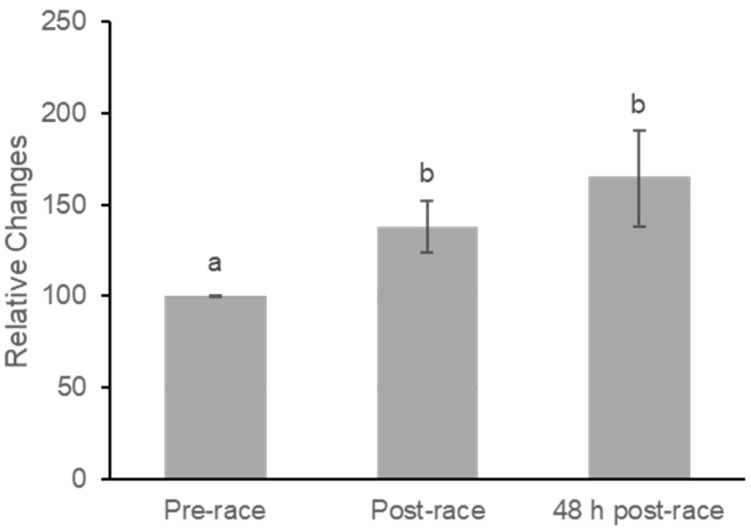
Relative Concentration Changes in Plasma Lycopene at Pre-race, Post-race, and 48 h Post-race. Values are mean ± SE. Plasma lycopene increased significantly from pre-race to post-race (*p* = 0.018) and remained elevated at 48 h post-race compared with pre-race (*p* = 0.021). No significant difference was observed between post-race and 48 h post-race values (*p* = 0.126). Superscript letters denote statistically significant pairwise differences based on least significant difference (LSD) post hoc testing; bars that do not share a letter differ significantly (*p* < 0.05).

Lycopene concentrations increased significantly following the marathon (Figure 3). Mean plasma levels rose from 0.133 ± 0.014 at baseline to 0.175 ± 0.020 immediately post-race and remained elevated at 0.181 ± 0.020 at 48 h post-race. A repeated-measures ANOVA indicated a moderate effect of time on plasma lycopene (F(2,40) = 5.43, *p* = 0.008; partial η^2^ = 0.21).

Pairwise comparisons indicated significant increases from pre-race to post-race (mean difference = −39.37 ± 15.29, *p* = 0.018), representing a moderate within-subject effect (Cohen’s dz = 0.56) and from pre-race to 48 h post-race (mean difference = −67.62 ± 26.96, *p* = 0.021), also corresponding to a moderate effect (dz = 0.55). Lycopene did not change significantly between post-race and 48 h post-race (*p* = 0.126), and the associated effect size was small to moderate (dz = 0.35).

### 3.3. Plasma Carotenoids: β-Carotene

**Figure 4:**
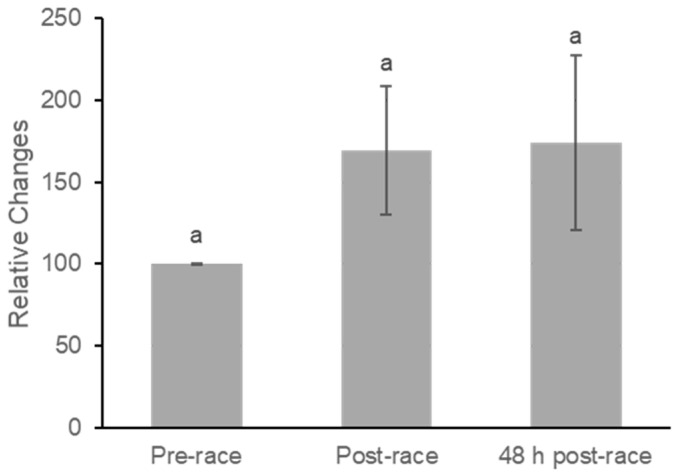
Relative Concentration Changes in All-trans Plasma B-carotene at Pre-race, Post-race, and 48 h Post-race. Values are mean ± SE. Repeated-measures ANOVA showed no significant differences in plasma all-trans β-carotene concentrations across time points. Pairwise comparisons indicated no significant change from pre-race to post-race (*p* = 0.099), from pre-race to 48 h post-race (*p* = 0.194), or from post-race to 48 h post-race (*p* = 0.894). Superscript letters reflect statistical comparisons based on least significant difference (LSD) post hoc testing; because no significant differences were observed, all bars share the same letter (*p* > 0.05).

Plasma β-carotene concentrations did not change significantly across the three time points (Figure 4). Mean values increased from pre-race to immediately post-race and remained similar at 48 h post-race. Repeated-measures ANOVA revealed no significant effect of time (F(2,40) = 2.07, *p* = 0.139), with a small effect size (partial η^2^ = 0.09).

Pairwise comparisons indicated a nonsignificant increase from pre-race to post-race (mean difference = −74.56 ± 43.07, *p* = 0.099), corresponding to a small-to-moderate within-subject effect (Cohen’s dz = 0.38). Comparisons between pre-race and 48 h post-race (dz = 0.29) and between post-race and 48 h post-race (dz = 0.03) demonstrated small to negligible effects, consistent with the absence of a meaningful acute response of plasma β-carotene within the 48 h recovery period.

### 3.4. Dietary Carotenoids

Dietary intake of β-carotene and lycopene remained consistent across the four time points. There were no significant differences between T1, which was 24 h before the race, T2 on the day of the race, T3, which was 24 h after the race, and T4, which was 48 h after the race. All pairwise comparisons were nonsignificant for both carotenoids (Table 2 and Table 3).

Body mass and total body water measured using bioelectrical impedance analysis (InBody) changed significantly across time. Body mass decreased from 159.8 ± 31.0 lb pre-race to 154.5 ± 28.9 lb immediately post-race (*p* < 0.001) and partially recovered to 158.4 ± 30.0 lb at 48 h post-race, remaining slightly lower than baseline (*p* = 0.031). Total body water also changed significantly across time (F(2,46) = 4.39, *p* = 0.018), with no significant difference between pre-race and immediately post-race values, but a significant reduction at 48 h post-race compared with post-race (*p* = 0.011).

## 4. Discussion

The purpose of this study was to examine the short-term effects of a marathon on the distribution and concentration of carotenoids in the plasma and skin of endurance runners. Our results demonstrated an acute, significant decrease in skin carotenoid (SC) levels immediately post-race, followed by a rebound above baseline levels at 48 h, paired against a tendency toward increased plasma lycopene concentrations (Figure 2 and Figure 3). These contrasting results suggest dual roles during extreme metabolic stress using circulation as an antioxidant mobilization pathway and the skin as a potential reactive antioxidant reservoir [16].

The most pronounced finding was the acute and significant depletion of SC levels immediately following the marathon (Figure 2). This change directly correlates with the high metabolic stress imposed by the event. Intense and prolonged exercise generates substantial metabolic stress and increases the production of reactive oxygen species (ROS) and other oxidative stress markers, which can lead to muscle fatigue and damage [6,17]. Carotenoids, including lycopene and β-carotene, are essential components of the antioxidative system, functioning to neutralize ROS, and can be stored in human skin [18]. The kinetics of carotenoid degradation following acute stress factors are known to be relatively fast, reaching maximal degradation within a number of hours [18]. The degradation observed in the skin likely reflects stored carotenoids being consumed in their protective role during metabolic stressors, such as competing in a marathon. As the skin acts as a major storage compartment [18,19,20], the large post-race drop in total SC suggests a rapid use of these antioxidant compounds to combat systemic and local oxidative load (Figure 2).

In contrast to the SC reduction, plasma lycopene levels demonstrated a trend toward an increase immediately following the race (Figure 3), while plasma β-carotene remained stable (Figure 4). This elevation in plasma concentrations is attributable primarily to the profound fluid shifts characteristic of intense physical activity, known as hemoconcentration [21,22,23]. Intense exercise causes plasma to shift from the intravascular space into the interstitial space, leading to an increased measured concentration of non-diffusible blood components, including plasma carotenoids, during higher-intensity exercise [11,22,24].

As carotenoids are lipophilic and stored predominantly in adipose tissue [11,20,25], their mobilization into the systemic circulation may contribute to elevated plasma concentrations. Prolonged endurance activities rely heavily on adipose tissue triglycerides as fuel, and the increased rate of lipolysis that occurs during a marathon releases fatty acids, with the possibility of co-mobilizing the fat-soluble carotenoids at the same time [16,25,26].

It is important to note that different methodological approaches were used to quantify carotenoids in this study. High-performance liquid chromatography remains the gold standard for analyte-specific plasma carotenoid quantification [1], whereas reflection spectroscopy provides a validated, non-invasive assessment of total dermal carotenoid status [10,11,12,13,14]. These methods assess distinct biological compartments and are not expected to yield directly comparable values due to differences in carotenoid storage and turnover kinetics [16,18,19]. A strength of the present study is the concurrent assessment of dermal and plasma carotenoids, which allows compartment-specific responses to prolonged endurance exercise to be examined.

The rebound in skin carotenoid levels observed at 48 h post-race, which exceeded baseline measurements, suggests a rapid restoration process. While blood serves as the transport medium for carotenoids [16,17,19,20], the skin acts as a storage depot [18,19,20]. Although recovery following degradation is generally a more prolonged effect, requiring several days before concentration levels stabilize [16,18], the quick replenishment and overshoot of SC we observed in 48 h suggests a more efficient process to a higher degree of degradation, such as from metabolic stress during a marathon (Figure 2). It is possible that once the marathon stress ended, the body rapidly restored its primary peripheral antioxidant barrier, drawing from the circulating pool (which showed a persistent trend of elevated lycopene) (Figure 3).

### Study Limitations

Our study is not without limitations, and the interpretation of these results warrants consideration from the following: First, while we were able to recruit an equal representation of males and females, our study’s participants were limited to 24 healthy endurance runners, consisting primarily of trained or highly trained athletes. This relatively small and homogeneous cohort limits statistical power and reduces the generalizability of our findings to broader or less-trained populations.

Second, plasma carotenoid values were uncorrected for plasma volume shifts. Since hemoconcentration is a significant consequence of intense exercise due to fluid loss, the observed increases in plasma lycopene may primarily reflect the volume shifts rather than an absolute increase in the carotenoid mass resulting from mobilization. The absence of concurrent measures (e.g., hemoglobin or hematocrit) to correct for fluid loss complicates the interpretation of the precise change in plasma concentration.

Third, the measurement of dermal concentrations via reflection spectroscopy (RS), while non-invasive and validated, yields a total measure of stored carotenoids. This contrasts with the targeted HPLC analysis used for plasma, which prevents direct comparisons between individual dermal carotenoids (e.g., lycopene vs. β-carotene) and masks which specific carotenoids contributed most significantly to the observed SC drop and rebound.

Fourth, the assessment of nutrient consumption relied on self-reported 24 h recalls (ASA24), a subjective method that is inherently prone to measurement error and participant bias.

Finally, substantial inter-individual variability in carotenoid absorption, metabolism, and tissue distribution is well documented and may have masked true physiological responses to marathon running, particularly for plasma carotenoids, despite the use of a repeated-measures design.

## 5. Conclusions

The findings of this study show a clear response of SC to the metabolic stressors of running a full marathon, with rapid uptake of dermal carotenoids and transient changes in systemic concentrations. The acute drop in SC levels reflects the rapid utilization of these antioxidants in response to the high levels of oxidative stress and free radicals generated by the intense exercise. The elevated plasma lycopene concentration post-race is likely indicative of hemoconcentration. The rapid rebound and transient elevation of skin carotenoid levels observed 48 h after the marathon indicate a dynamic recovery of dermal carotenoid status during early recovery.

Future research should address our study’s limitations by implementing plasma-volume correction methods to accurately distinguish between the effects of hemoconcentration and the actual net mobilization of carotenoids from adipose stores. Ultimately, our findings can optimize training, hydration, and nutritional strategies aimed at supporting the antioxidant capacity of a recovery phase following extreme training or events.

## Figures and Tables

**Table 1:** Participant demographic and training characteristics.

Characteristic	Total (*n* = 24)	Males (*n* = 12)	Females (*n* = 12)
Age (years)	37 ± 10	39.3 ± 11.8	36.6 ± 8.2
Height (m)	1.70 ± 0.10	1.81 ± 0.07	1.64 ± 0.06
Body mass (kg)	70.1 ± 14.0	79.6 ± 8.8	60.6 ± 9.4
Marathon finish time (min)	271.9 ± 60.5	270.6 ± 50.4	273.1 ± 69.9
Years of running experience (years)	11 (3–30)	12 (3–30)	10 (3–25)
Full marathons completed, *n*	4 (0–133)	5 (0–133)	3 (0–40)
Weekly running mileage (miles/week)	24 ± 13	25 ± 14	23 ± 12
Training classification, *n*			
– Recreationally active	6	3	3
– Trained	15	7	8
– Highly trained	3	2	1

Values are presented as mean ± standard deviation or median (range), as indicated.

**Table 2:** β-carotene intake across four time points and pairwise comparisons.

Time Point	Mean ± SE (µg)	T1 *p*-Value	T2 *p*-Value	T3 *p*-Value	T4 *p*-Value
T1 (24 h before race)	5674.53 ± 10818.48	—	0.300	0.596	0.304
T2 (day of race)	3154.71 ± 3666.62	0.300	—	0.614	0.946
T3 (24 h after race)	4203.58 ± 8077.55	0.596	0.614	—	0.193
T4 (48 h after race)	3034.34 ± 6128.82	0.304	0.946	0.193	—

Values are mean ± standard error (SE). Pairwise *p*-values are based on least significant difference (LSD) post hoc comparisons among the four time points.

**Table 3:** Lycopene intake across four time points and pairwise comparisons.

Time Point	Mean ± SE (µg)	T1 *p*-Value	T2 *p*-Value	T3 *p*-Value	T4 *p*-Value
T1 (24 h before race)	9592.88 ± 22,768.72	—	0.333	0.124	0.279
T2 (day of race)	4637.96 ± 5255.80	0.333	—	0.422	0.460
T3 (24 h after race)	3347.75 ± 7175.22	0.124	0.422	—	0.846
T4 (48 h after race)	3727.97 ± 4991.47	0.279	0.460	0.846	—

Values are mean ± standard error (SE). Pairwise *p*-values are based on least significant difference (LSD) post hoc comparisons among the four time points.

## Data Availability

The original contributions presented in this study are included in the article/Supplementary Material. Further inquiries can be directed to the corresponding author.

## Supplementary Materials

The following are available online at https://www.mdpi.com/article/10.3390/nu18030437/s1. Raw participant data: Intake carotenoid.xlsx, Skin Carotenoid Data 2024.xlsx, Plasma Carotenoid HPLC data summary Final.xlsx, Plasma Carotenoids.sav, Skin Carotenoids.sav, Marathon IRB.pdf, Body mass and Total water.spv, InBody.sav.
