# Supplementary material for: Effects of Marathon Running on Skin and Plasma Carotenoids in Endurance Runners
Source: Nutrients. 2026 Jan 29;18(3):437. doi: 10.3390/nu18030437 (PMC12899393; doi:10.3390/nu18030437)
Supplement: Supplementary file 1 [file nutrients-18-00437-s001.zip › Marathon Study/Marathon IRB.pdf]

Date: 12-14-2025

IRB #: IRB-AY23-24-173
Title: Ogden Marathon Study
Creation Date: 11-20-2023
End Date:
Status: Approved
Principal Investigator: Saori Hanaki
Review Board: Ad Hoc Review Board
Sponsor:

Study History

Submission Type Initial	Review Type Expedited	Decision Approved
Submission Type Modification	Review Type Expedited	Decision Approved
Submission Type Modification	Review Type Expedited	Decision Approved

Key Study Contacts

Member Stacie Wing-Gaia	Role Co-Principal Investigator	Contact swinggaia@weber.edu
Member Damon Joyner	Role Co-Principal Investigator	Contact damonjoyner@weber.edu
Member Bryan Dowdell	Role Co-Principal Investigator	Contact bryandowdell@weber.edu
Member David Aguilar-Alvarez	Role Co-Principal Investigator	Contact daguilaralvarez@weber.edu
Member Saori Hanaki	Role Principal Investigator	Contact saorihanaki@weber.edu
Member Bryan Dowdell	Role Primary Contact	Contact bryandowdell@weber.edu

Initial Submission

1- Getting Started

Weber State University IRB

Welcome and Thank You for your interest in completing human subjects research at or in association with Weber State University!

Please keep the following in mind as you complete your application:

- Incomplete submissions will not be reviewed.
- You cannot begin data collection until a formal approval letter from the IRB has been received.
- The IRB meets as needed during the academic year. Please submit the application as soon as possible and allow at least 7 days for an expedited review and 30 days for a Full Board Review.

About Cayuse IRB Software

Cayuse IRB is an interactive web application. As you answer questions, new sections relevant to the type of research being conducted will appear on the left-hand side. Therefore not all numbered sections may appear. You do not have to finish the application in one sitting. All information can be saved.

Additional information has been added throughout the form for guidance and clarity. That additional information can be found by clicking the question mark it the top-right corner of each section.

For more information about the IRB submission Process, IRB Tracking, and Cayuse IRB Tasks, please refer to the Cayuse IRB Procedures Manual.

Getting Started

Throughout the submission, you will be asked to provide the following (as applicable):

- Detailed Study Information
- Informed Consent Forms (in appropriate languages)
- Study Recruitment Documents (flyers, social media posts, recruitment scripts, etc)
- Copies of CITI training certificates for all study personnel
- All instrument, questionnaires, surveys, interview questions, discussion questions
- Letters of support from any sponsoring or supporting institutions/organizations
- Photographic/Video Release from research participants.

*required

IRB Subcomittee

Please indicate the most appropriate Weber State IRB Subcommittee to review your research project.

NOTE: In nearly all instances this is the College the PI is employed.
CCEL, the Library and other campus entities existing outside of a College should select AD Hoc Review.

College of Arts & Humanities

School of Business & Economics

✓ College of Education

College of Engineering, Applied Science & Technology

College of Health Professions

College of Science

College of Social and Behavioral Sciences

*required

Researcher Agreement

This research study involves the use of human subjects. I understand the university's policy concerning research involving human subjects and by submitting this application I agree to:

- Obtain voluntary and informed consent of subjects who are to participate in this project.
- Report to the IRB any unanticipated effects on subjects which become apparent during the course of, or as result of, the experimentation and the actions taken.
- Cooperate with members of the committee charged with continuing review of this project.
- Obtain prior approval from the committee before amending or altering the scope of the project or implementing changes in the approved consent document.
- Maintain the documentation of consent forms and progress reports as required by institutional policy for three years.
- Safeguard the confidentiality of research subjects and the data collected when the approved level of research requires it.

I have read the information above and I am ready to begin my submission.

✓ Yes

2- Submission Information

*required

What type of activity is this submission for?

✓ Research Study

Quality or Process Improvement Project - Medical or Health Related

Quality or Process Improvement Project - NON Medical or Health Related

Clinical Trial

Single Patient, Treatment Use, Continued Access Drug/Device Study

Emergency (or Compassionate) Use of Investigational Drug or Device

Student Directed Classroom-Based Research

*required

Is this a multi-institutional study?

Yes

✓ No

*required

Research Categorization

Do you believe the research in this application meets the criteria for exemption?

Yes

✓ No

*required

Select the level of review you believe is appropriate for this research

✓ Expedited - low to moderate risk

Full Board - moderate to high risk; research involving protected status groups or individuals

3- Study Information

*required

What is your status at Weber State University?

- ✓ Faculty
- Student
- Staff
- Other

Study Personnel

Note: If you cannot find a person in the people finder, please contact the IRB Office.

*required

Principal Investigator

*Provide the name of the Principal Investigator of this study. This individual is in charge of the research team and must be a Weber State employee (Faculty or Staff). Students may **NOT** serve as a sole PI, Graduate students may serve as a CO-PI, undergraduate students may be on the research team but may **NOT** serve as a PI.*

Name: Saori Hanaki
Organization: Exercise and Nutrition Science
Address: 1435 Village Dr DEPT 2805 , Ogden, UT 84408-2805
Phone: 8016266626
Email: saorihanaki@weber.edu

Co-Principal Investigator(s)

Provide the name(s) of Investigator(s) for this study.

Name: Stacie Wing-Gaia
Organization: Exercise and Nutrition Science
Address: 1435 Village Dr DEPT 2805 , Ogden, UT 84408-2805
Phone: 8016268942
Email: swinggaia@weber.edu

Name: Damon Joyner
Organization: Exercise and Nutrition Science
Address: 1435 Village Dr DEPT 2805 , Ogden, UT 84408-2805
Phone: 8016266627
Email: damonjoyner@weber.edu

Name: Bryan Dowdell
Organization: College of Education
Address:
Phone:
Email: bryandowdell@weber.edu

Name: David Aguilar-Alvarez
Organization: Exercise and Nutrition Science
Address: 1435 Village Dr DEPT 2805 , Ogden, UT 84408-2805
Phone: 8016268867
Email: daguilaralvarez@weber.edu

*required

Primary Contact

Provide the name of the Primary Contact of this study. This person does **NOT** have to be the PI and should be the person responsible for corresponding with the IRB and the investigative team.

Name: Bryan Dowdell
Organization: College of Education
Address:
Phone:
Email: bryandowdell@weber.edu

Other Personnel - WSU Affiliated

Provide the name(s) of ***ALL*** *other personnel for this study. Anyone who will have direct contact with participants and/or access to identifiable participant data.*

Other Personnel - NON WSU Affiliated

Provide the name(s) of ***ALL*** *other personnel* ***NOT AFFILIATED*** *with WSU (individuals without WSU log in credentials) for this study. Anyone who will have direct contact with participants and/or access to identifiable participant data.*

-Please provide Name, Affiliation and Email address.

The following staff/faculty at Weber State will also be co-investigators (their names are not in this system for whatever reason):

- Qi Jin, assistant professor in Exercise and Nutrition Sciences (ENS), qijin1@weber.edu
- Jamie Stein, instructor in ENS, jamiestein@weber.edu
- Sara Phlypo, ENS Lab coordinator, saraphlypo1@weber.edu

The following non-WSU affiliated individual will also be assisting with data collection and analysis for graduate-level research experience:

- Anthony Ludwig, graduate student at SJSU, anthony.f.ludwig@gmail.com

*required

Study Site

Please select all sites at which research will be conducted.

✓ Weber State University

At which Weber State University facilities will this research take place. (select all that apply)

Main Campus - Ogden

West Center

Farmington Station

Community Education Center - Ogden

Center for Continuing Education - Clearfield

Davis

Morgan Center

Other WSU affiliated site(s)

✓ External Site (non Weber State University)

Please provide the names of the external collaborating sites.

Some data collection will be conducted in our reserved sites near the finish line and vendors of the Ogden Marathon Expo in downtown Ogden. Blood and urine samples will be stored/analyzed in the Nutrition biochemistry lab in Swenson Hall.

Study Dates

Please provide the PROJECTED study start and end dates. Projects may not begin until approval letter is received.

*required

Start Date

01-01-2024

*required

End Date

12-31-2024

Departmental Approval

*required

Does your Department require approval of IRB submissions?

✓ Yes

*required

Please upload a signed departmental approval form.

[Download Form](#)

[Departmental Form IRB Ogden Marathon.pdf](#)

No

4- Subject Information

Subject Enrollment

Enter the number of subjects that will be enrolled in this study.

*required

Total Study Enrollment

Please enter the total number of subjects to be enrolled at all study sites.

50. We are enrolling 20 subjects for the full protocol. We would like to recruit 30 more subjects for a partial protocol (these subjects would only complete surveys/questionnaires).

*required

Enrollment at Weber State University

Please enter the number of subjects that will be enrolled at ***Weber State University****.*

0-50

*required

Ages

Select the age range of subjects that will be enrolled in this study. Check all that apply.

Fetus

Birth to less than 1 month

1 month to less than 12 years old

12 years old and less than 18 years old

✓ 18 years and older

*required

Vulnerable Populations

*Please check the population(s) that will be **TARGETED** for enrolled. Check all that apply.*

Fetuses

Pregnant Women

Minors with Parental Consent

Minors who can Consent Themselves (emancipated minors, minors in states that allow consent; please direct any questions about this to the IRB office)

Prisoners

Individuals with Cognitive Impairments

Economically Disadvantaged Persons

Educationally Disadvantaged Persons

Other

✓ None of the Above

5- Study Design

*required

Research Summary and Justification

Please provide a detailed summary that includes background and justification for the proposed research.

Please limit this section to 1000 words, citations should be included as a separate Appendix if necessary.

This research proposal is inherently an extension of our department's (Exercise and Nutrition Sciences; ENS) recently awarded Ambrose Amos Shaw Endowed Chair Award. This was awarded to promote a collaborative research opportunity for faculty and students alike to investigate characteristics and biomarkers in marathon runners.

Marathon running induces considerable physiological, psychological, and biomechanical strain on participants (Sanchez, Corwell, & Berkoff, 2006). The duration of exercise required to complete a marathon increases biological markers of cardiac stress (e.g. Troponin T), inflammation (e.g. TNF-α), and intestinal permeability (e.g. Fatty Acid Binding Protein) (Bernat-Adell et al., 2019; Da Ponte et al., 2018; Karhu et al., 2017; Scherr et al., 2011). Further, nutritional practices leading up to and during a marathon have considerable impacts on performance and recovery (Almond et al., 2005; Mielgo-Ayuso et al., 2020). The repetitive eccentric contractions occurring during running combined with the long distance (26.2 miles), provides the perfect scenario to induce considerable muscle damage (Clarkson, 2007; Del Coso et al., 2013; Kyröläinen et al., 2000). As such, many biological and biomechanical markers representing this damage are elevated following a marathon (Del Coso et al., 2013; Mielgo-Ayuso et al., 2020). This may have impacts on many biomechanical properties that affect gait, stride length, stride rate, running economy, and ultimately function (Del Coso et al., 2013; Kyröläinen et al., 2000). Muscle damage not only leads to biomechanical changes and soreness, but also challenges other physiological systems to maintain function. For example, certain cellular contents released from damaged muscle fibers (e.g. myoglobin) into the circulation can have nephrotoxic effects (i.e. damage the kidneys), which may compromise overall renal function (Bosch, Poch, & Grau, 2009; Clarkson, 2007). When running a marathon, the renal system is stressed due to reduced perfusion associated with exercise and is often combined with hypovolemia from dehydration (Mansour et al., 2017; Sanchez et al., 2006). Therefore, adding a toxic substance such as myoglobin, damages the nephrons and leads to elevations in biological markers of acute kidney injury post-race (e.g. creatinine, kidney injury molecule-1) (Mansour et al., 2017; McCullough et al., 2011; Mingels, Jacobs, Kleijnen, Wodzig, & Dieijen-Visser, 2009). The responses to marathon running are variable in the literature, due to differences in weather, altitude, and course design. This variation is beneficial for providing a wide response pattern and allows for a personalized assessment for expected results given specific conditions.

As such, the purpose of this study is to evaluate nutritional behaviors and the biomechanical and physiological responses to running the Ogden Marathon. This marathon course contains a considerable downhill component, which facilitates muscle damage more-so than flat marathons. Therefore, we expect considerable physiological and biomechanical strain that may lead to delayed recovery. Further, we intend to assess variables pre-marathon, immediately post-marathon, and at 48 hours post-marathon. Unfortunately, relatively few studies have evaluated the effects of completing a marathon past 24 hours, likely due to participant availability. The relative lack of data for responses past 24 hours is concerning as

many biological markers do not peak until as late as 72 hours (e.g. creatine kinase) or may have variations that peak immediately and then again after several days (e.g. kidney injury molecule 1). Thus, we propose to investigate these variables at various time points to contribute to a needed area in research.

Appendix

Please attach appendix document(s) as needed.
Appendix A - References.docx

*required
Hypothesis

Provide the study hypothesis.
We hypothesize that variables associated with muscle damage will remain elevated at the 48-hour follow-up, however only certain physiological variables (e.g. kidney injury molecule-1) will be increased following recovery.

*required
Objectives

Provide the study objectives.
The Ogden Marathon is unique in that its course has a considerable downhill component. This uniqueness allows us to analyze and contribute to the growing body of variable response patterns for various marathons.

- Assess baseline biomarkers as well as up to 48 hours post-marathon for analysis.
- Analyze health patterns, biomechanics, and nutrition habits to further explore potential covariates and relationships.

*required
Outcome Measures

Provide the main study outcome measures/dependent variables.

We will mainly be assessing muscle damage biomarkers and hydration markers via blood and urine samples at various time points. We will also be collecting the following outcome measures to assess further relationships:

- Scales for thirst perception (detailing the level or sensation of thirst), muscle pain (how much pain they are feeling), and fatigue (how tired they feel) to evaluate the perceptual strain.
- Body weight and composition before and after the race
- Skin carotenoid levels before and after race
- Training/medical history
- Questionnaires detailing participant NSAID use will be completed before, following, and after 48 hours of recovery.
- Dietary patterns such as caloric intake and nutrient breakdown
- Run/stride frequency during race
- Isometric strength before and after race

*required

Inclusion Criteria

List and describe the inclusion criteria.

- Participants to be between the ages of 18–65 years old
- Participants are already registered and running the full Ogden Marathon

*required

Exclusion Criteria

List and describe the exclusion criteria.

Exclusion Criteria is only for the full protocol:

- No known cardiovascular, respiratory, gastrointestinal bleeding, inflammatory, metabolic/ fluid-electrolyte disorder or other chronic diseases, or pregnant
- Prone to vasovagal syncope as a result of venipuncture
- Not able to attend all visits

6- Study Procedures

*required

Describe all study procedures.

Provide a complete and thorough description of the procedures in the proposed research.

This description should encompass the experimental course of a subject from their entry into the study to their completion of the study.

This study's protocol is multi-faceted. We will recruit subjects through Ogden's GOAL (Get Out And Live) Foundation whom we have already been approved and partnered with for race day logistics (see attached MOU in the Appendix). This study will consist of a familiarization and three visits (pre-race expo, race day, and 48-hour follow-up). The familiarization (in-person or virtual Zoom) will include completing the informed consent form, followed by dietary surveys (Appendix) and a 24 h history questionnaire (Appendix) The participant will also be enrolled in a Strava group with de-identified codes. Following completion of thes forms, demographic information (e.g. height, weight, age, gender, and ethnicity) will be collected. Meeting times will then be scheduled for the day before the race (at the expo) and the morning of the race.

During visit one at the expo, participants will also be instructed to record dietary intake for prior to the race, during the race day, and following the race using the automated self-administered 24 hr recall (ASA24-202 Appendix). The participants will be provided a login specific to their participant ID, but deidentified to protec their identity. Participants will also complete a training/health history survey and body weight/composition assessment. Lastly, they will be given a urine sample collection kit and further instructions, checks, and reminders for race day.

On the day of the race (visit two), the participant will report to the designated area near the shuttle location that will transport them to the race start line. Upon arrival, participants will be asked to provide the urine sample in a urine specimen cup and have their body mass measured on a standard research body mass scale. Hydration will be analyzed using urine specific gravity via refractometer, urine color, and urine osmolality via freezing point depression (Advanced Instruments osmometer). Urine will also be stored for la analysis of biological markers (i.e. inflammatory markers, renal biomarkers, cytokines, immune markers, markers of metabolism, cardiovascular markers). Participants will place their hand on our carotenoid senso to measure skin carotenoid levels. Participants will also complete a pre-race questionnaire (Appendix), pair fatigue, and thirst scales (Appendix) as well as record body weight/body composition through our InBody scale. They will also be tested for isometric leg strength on both legs with a portable force transducer. A sm subset of runners (n=10) will also be instrumented with a RunScribe device on their shoe that will track biomechanical information. A small blood sample (~20 mL or ~1.4 tablespoon/one tube) will be obtained vi venipuncture from the antecubital vein from trained phlebotomists. Blood samples will be used to analyze osmolality and saved for later analysis of biological markers (i.e. inflammatory markers, renal biomarkers, cytokines, immune markers, markers of metabolism, cardiovascular markers). Participants will then be provided a wristband or stamp that will indicate their participation for identification after the race by researchers. They will then go complete the race.

Upon finishing the race, the participants will be asked to report to the data collection tent near the finish line Upon reporting the participant will provide a urine sample, body mass/composition measurement, and bloo sample via venipuncture from the antecubital vein (~20 ml or one tube). Participants will also complete a post-race questionnaire (Appendix), pain, fatigue, and thirst scales, and provide a description of their dietar intake during the race. They will also be re-tested for isometric leg strength on both legs. The runners wear

the RunScribe units will also be de-instrumented. Participants will then be provided beverages and snacks desired) and they will schedule a time to come to their 48-hour follow-up visit at the Weber State University Human Performance Laboratory. Participants will continue to record dietary intake using the ASA24-2022 f an additional 2-days post-race.

Upon arriving for the 48-hour follow-up visit (visit three), participants will provide a urine sample, body mass measurement, and blood sample via venipuncture from the antecubital vein (~20 ml or two tubes). Participants will also complete a recovery questionnaire (Appendix J), pain, fatigue, and thirst scales. They will also be tested for isometric leg strength on both legs. Participants will also be instructed to change into tight fitting clothing (e.g. spandex shorts, sports bra, bathing suit) to complete a BodPod assessment of boc composition. Once complete the participants will be provided a copy of their BodPod results.

For participants who elect to partake in the partial protocol, they will only have to complete the familiarizatic and complete the online ASA24 dietary recalls on the same days as the full protocol.

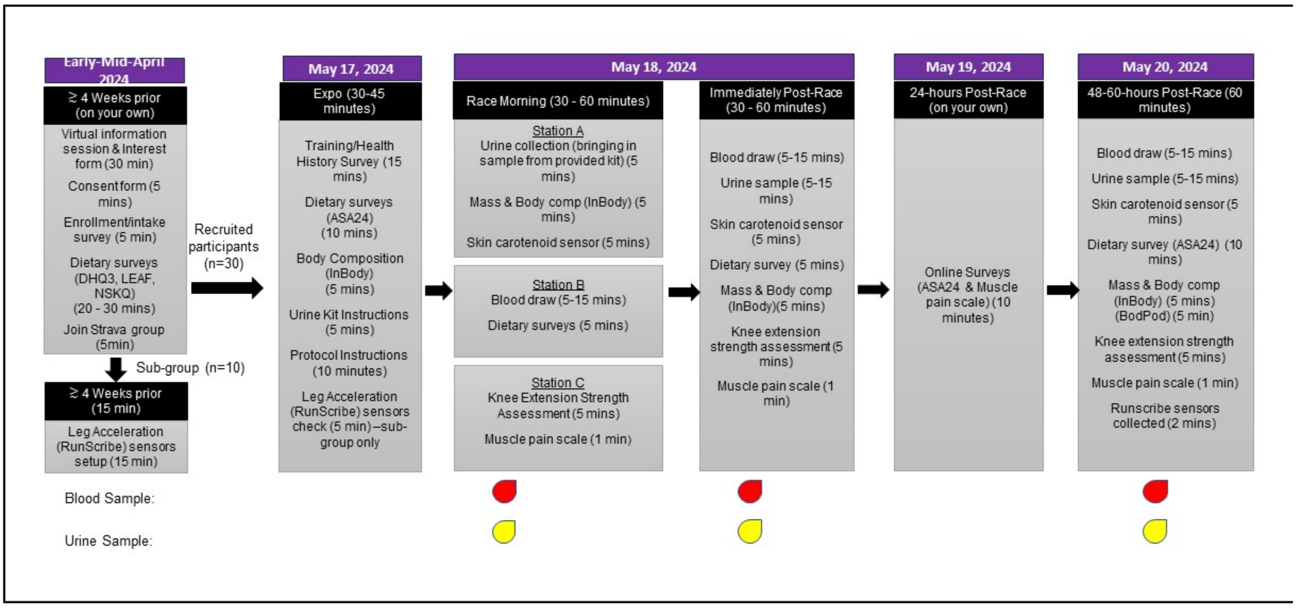

*required

Describe your subject recruitment procedures and any material inducements given for participation

Recruitment will occur via word of mouth, emails sent to race participants, and social media postings such as Facebook pages. Recruitment statements will read as follows: Participants needed for study runners completing Ogden Marathon.

- Participants (up to 20) for the full research protocol including recording your diet and providing urine and blood samples can receive a VIP shuttle to the marathon starting line, report of body composition via BodPod, and $100 gift card after completing the study.
- Additional participants are sought to completes surveys only (no urine and blood samples). The survey-only participants are not eligible for the gift card or a body composition analysis.

In order to participate, you must be already registered for and run the Ogden Full Marathon and not pregnant. If interested, please visit to this link (link to an online interest form). If you have any questions, contact Dr. Saori Hanaki at saorihanaki@weber.edu, Dr. Stacie Wing-Gaia at swinggaia@weber.edu, Dr. Bryan Dowdell at bryandowdell@weber.edu, Dr. Damon Joyner at damonjoyner@weber.edu, Dr. Qi Jin at qijin1@weber.edu, or Dr. David Aguilar Alvarez at daguilaralvarez@weber.edu.

*required

Describe the duration of study participation, the length and number of study visits, and the timetable for study completion.

Please be sure to provide the following:

- How many times human subjects will perform research activities;
- How long each visit will take;
- Total time requirement for a subject to complete the proposed research?

Participants will complete a familiarization visit and three additional visits (pre-race expo, race day, and 48-hour recovery day). The familiarization should take ~30 minutes. The pre, post, and 48 hour recovery visits will take ~30 minutes. The surveys completed on their own take ~15 minutes each. Therefore total time commitment is ~3 hours.

*required

Describe the information to be gathered and the means for collecting and recording data.

Many studies use multiple instruments/questionnaires/surveys as part of the research methodology. If applicable to the proposed research, list each instrument/questionnaire/survey that will be administered to subjects and provide a rationale for the inclusion of each one.

If previously collected data is to be used, describe both the previous and proposed uses of these data.

- Hydration will be assessed via a refractometer, urine color chart, and osmometer from urine samples.
- Blood biomarkers associated with muscle damage will be assessed via assay kits.
- Scales for thirst perception (detailing the level or sensation of thirst), muscle pain (how much pain they are feeling), and fatigue (how tired they feel) to evaluate the perceptual strain.
- Body weight and composition via Inbody scale and Bod Pod.
- Skin carotenoid levels via skin carotenoid sensor.
- A training/medical history questionnaire will be used to evaluate health history and quantify the level of activity in which individuals regularly engage.
- Questionnaires detailing participant NSAID use will be completed before, following, and after 48 hours of recovery.
- A validated online diet recording survey (ASA24) as well as DHQ3, LEAF, NSKQ will be used to quantify macronutrient and micronutrient intake for the duration of the study.
- RunScribe sensors will be attached to select participants' shoes to identify their biomechanical responses.
- Isometric strength will be assessed using a force transducer to identify changes associated with muscle damage.

Study Instruments

Attach all instruments (i.e. personality scales, questionnaires, evaluation blanks, etc) to be used in the study.

ACSM Health History & Physical Activity Questionnaire.pdf

Nutrition Knowledge Survey.docx

McKay Training Status Classification.pdf

The LEAF-Questionnaire.pdf

Sample questionnaire DHQ3.pdf

ASA24 Info.docx

Ogden Marathon Data Collection Checklist.docx

*required

Survey, Questionnaire, or Interview

Will the study utilize surveys, questionnaires, or interviews?

✓ Yes

*required

Attach all copies of surveys, questionnaires, or interviews.

If your survery is hosted on an online application, WSU IRB prefers a link to the study. Please use the attached link function.

ACSM Health History & Physical Activity Questionnaire.pdf

Nutrition Knowledge Survey.docx

McKay Training Status Classification.pdf

The LEAF-Questionnaire.pdf

Sample questionnaire DHQ3.pdf

ASA24 Info.docx

Ogden Marathon Data Collection Checklist.docx

No

*required

Will the survey, questionnaire, or interview record any information that can identify the participants?

Yes

✓ No

*required

Genetic Testing

Will this study involve genetic testing?

Yes

✓ No

*required

Drugs, Devices, Biologics

Will the study involve administering any of the following? Check all that apply.

Drug

Biologic

✓ Device

*required

Please describe.

A RunScribe motion sensor device will be placed on a subset of the subject's shoes during the race.

None of the above

*required

Participant Data, Specimens, and Records

Does this project involve the collection or use of materials (data or specimens) recorded in a manner that could identify the individuals who provided the materials, either directly or through identifiers linked to these individuals?

Yes

✓ No

*required

Specimen and Sample Retention

Does the research team plan to retain any Specimens or Biologic samples for future analysis.

✓ Yes

*required

Please explain the intent of the retention and expected destruction date.

NOTE: Intend to retain samples for future analysis **MUST** be clearly stated in your informed consent document and disclosed to participants.

All blood and urine samples will be properly stored in our biochemistry lab (SW 133) for future biomarker analysis. These samples will be retained for the maximal allowable time of three years after which all samples will be properly disposed of according to university and OSHA protocols.

No

7- Participant Protection

Risk: *Generally, risk assessment in research considers the harm, trauma, discomfort, stress, or any other undesirable or untoward consequence of being a research subject whether anticipated or unexpected. Risk may take the following forms: physical, psychological, emotional, financial, and/or social. This list is representative but not comprehensive.*

Please consider the following questions based on the experiences that subjects might encounter through participation in the proposed research.

*required

Potential Risk

Whether great or small, do you think there is potential risks to subjects that participate in the proposed research.

✓ Yes

No

Potential Risks

*required

Describe immediate risks, long-term risks, rationale for the necessity of such risks, alternatives that were or will be considered, and why alternatives may not be feasible.

There are multiple risks associated with this study. Most of these risks are inherent and cannot be avoided because of the purpose of the study, thus alternatives are not feasible.

The full protocol (includes blood/urine samples) risks are:

- Inherent risks associated with voluntary exercise and exertion
- Inherent risks associated with blood draws such as feelings of discomfort and pain associated with needles as well as vasovagal syncope (fainting upon sight of blood/needles)

- Subjects are required to answer questions from health surveys or questionnaires such as dietary recalls that may induce feelings of discomfort.
- We cannot guarantee absolute confidentiality

The partial protocol (only includes surveys/questionnaires) risks are:

- Subjects are required to answer questions from health surveys or questionnaires such as dietary recalls that may induce feelings of discomfort.
- We cannot guarantee absolute confidentiality

What steps will be taken by the investigator to reduce the aforementioned risks?

- Exclusion criteria of pre-existing conditions to mitigate inherent risks associated with exercise (anybody with CVD diseases, pulmonary conditions, etc cannot participate).
- Exclusion criteria of those weary of blood draws (i.e. prone to vasovagal responses such as fainting upon the sight of blood/needles during blood draws).
- We will be using trained phlebotomists for the blood draws. Universal precautions (PPE) will be utilized to minimize any chance of infection. The participants will be in a seated position during the blood draw (minimizing any injury should they feel lightheaded). Also, pressure will be applied to the blood draw sight to minimize soreness.
- Subjects will be reminded that this study is entirely voluntary. If they want to discontinue participating, they may drop out at any time and contact research personnel.
- All surveys and assessments will be completed by a trained research staff member.
- All data will be de-identified.

*required

Describe any potential legal, financial, social, or personal affects on subjects.

None

What steps will be taken by the investigator to reduce the aforementioned risks?

*required

Will deception be used as a method of data gathering?

Yes

✓ No

*required

Does this research require face to face contact between researchers and participants

✓ Yes

*required

Please detail your COVID 19 Mitigation Procedures.

If required by the university, the IRB, and/or the state of Utah, all subjects and researchers will wear masks (face coverings) at all times.

No

*required

Expected Benefits

Please describe any benefits that research subjects will receive as a direct result of their participation in the proposed research.

Note: *compensation is not considered a benefit*

Subjects will be given interesting information about their heath habits and body composition through assessments that normally cost money. In addition, subjects are given access to complimentary and private access to a bus shuttle and restrooms before and after the marathon designated only for study subjects.

Please describe how this research may provide benefit to scientific knowledge, a specific discipline, and/or society in general.

By participating and contributing to data collection, subjects can know that the findings from this research will contribute to a missing area of research in terms of marathon responses.

*required

Subject Compensation

---

*required

Will subjects receive any form of compensation for participation in this investigation?

Compensation includes but is not limited to: University Credit, extra points in a course, gift cards or any form of monetary instrument.

✓ Yes

No

*required

Please describe and detail any and all forms of compensation that subjects may receive.

$100 gift card

*required

Safeguarding Subjects' Identity

What uses will be made of identifiable information obtained from the subjects?

The only identifiable information (name) will be the signed informed consent form. Signed informed consent forms will be kept separated from data collected, and the signed/completed hard copy documents will be stored in a locked cabinet in our (ENS) lab coordinator's office (Sara Phlypo who is part of research personnel).

What precautions will be taken to safeguard identifiable records or individuals?

The only identifiable information (name) will be the signed informed consent form. Signed informed consent forms will be kept separated from data collected, and the signed/completed hard copy documents will be stored in a locked cabinet in our (ENS) lab coordinator's office (Sara Phlypo who is part of research personnel).

*required

Data Management

Protecting ***hard copy*** data may involve de-identification of data (see:NISTIR-8053) and secured storage locations and conditions (see: Weber State University PPM 10-1).

Describe what type of hard copy data will be generated by the proposed research (i.e.,

notes, audio/video tapes, questionnaires, etc.). Where will this hard copy data be stored and how will it be protected?

The only identifiable information (name) will be the signed informed consent form. Signed informed consent forms will be kept separated from data collected, and the signed/completed hard copy documents will be stored in a locked cabinet in our (ENS) lab coordinator's office (Sara Phlypo who is part of research personnel).

Hard copy surveys/questionnaires (such as the nutritional and dietary surveys) and the stored biologicals samples (in SW 133 biochemistry lab) will use de-identified codes (i.e. OMS-01). Hard copy data will be stored securely and separately from the informed consents in our lab coordinator's office. Biological samples will be stored securely in our -80 degree freezer in SW 133 biochemistry lab in which only authorized users have access.

Protecting ***electronic data*** may involve a secure network, password access, and data de-identification/ encryption (see: Weber State University PPM 10-1)

Describe what type of electronic data will be generated by the proposed research (i.e., computer files/ spreadsheets, questionnaires, images, video, audio/mp3 files, etc.) Where will this electronic data be stored and how will it be protected

---

All questionnaire results (de-identified) will be entered into data spreadsheets saved in a Box/Google Drive Folder which only the study investigators will have access to. These are password-protected and managed by the university. The ASA24 platform data are stored under a subject's ID number without any identifiable information. ASA24 platforms are password-protected and only the research personnel will have access to it. The exported data from this platform will be stored in the Google Drive/Box mentioned above.

*required

Will the research require accessing student educational records?

Yes

✓ No

*required

On what date will study data and materials with identifiable information be destroyed?

Please select a year and date.

12-31-2027

*required

Will raw data be made available to anyone other than the principal investigator and the immediate study personnel?

Yes

✓ No

*required

Informed Consent

Describe the procedures for obtaining informed consent.

Informed Consent will be obtained in either hard-copy form or virtually via a live Zoom session with recruited participants. We will have two separate Informed Consent forms: one for those interested in completing the full protocol (which includes blood draws and urine samples) and one for the partial protocol (which only includes the survey/questionnaires).

*required

Please attach the your Informed Consent Document(s). Please include all versions/languages you intend to utilize. **PDF format required!**

You will also have a chance to attach on the last page of the application.
Download Template (Word Doc Format)
Informed Consent Full Protocol.doc

Informed Consent Partial Protocol.docx

8- Conflict of Interest

*required

Do you or any investigator(s) participating in this study have a financial interest related to this research project?

Yes

✓ No

Study Funding

*required

Does this study have **EXTERNAL** funding

Yes

✓ No

*required

Does this study have an **INTERNAL** funding sources

✓ Yes

Please list sources of INTERNAL funding.

Ambrose Amos Shaw Endowed Chair Award

No

9- Attachments

Participant Protection

Informed Consent Form

Please upload **ALL** versions of the Informed Consent document that maybe used. If requesting a waiver please upload justification here. **PDF format required!**
Informed Consent Full Protocol.doc

Informed Consent Partial Protocol.docx

If requesting an Informed Consent Waiver/Alternation under 45 CFR 46.16 please explain.

*required
Research Human Subjects Training Certificate(s)

Please upload a CITI certificate for **EVERY** investigator listed on the application
Citi Certificate - Jamie Stein.pdf

CITI certificate Damon Joyner.pdf

CITI DavidAguilar.pdf

CITI_qijin.pdf

citiCompletionCertificate_Dowdell_2023.pdf

Stacie Wing-Gaia CITI Completion certificate.pdf

citiCompletionReportHanakiJan2021.pdf

citiCompletionCertificate_Sara Phlypo.pdf

Study Procedures

Study Documents

If applicable, this includes flyers used for recruitment.
Recruitment announcement.docx

Study Instruments

Attach all instruments (i.e. personality scales, questionnaires, evaluation blanks, etc) to be used in the study.
ACSM Health History & Physical Activity Questionnaire.pdf

Nutrition Knowledge Survey.docx

McKay Training Status Classification.pdf

The LEAF-Questionnaire.pdf

Sample questionnaire DHQ3.pdf

ASA24 Info.docx

Ogden Marathon Data Collection Checklist.docx

FDA Letter

Study Design

Appendix
Appendix A - References.docx

External IRB of Record

Study Protocol

Attach the protocol for this study that was reviewed by the Outside IRB.

Outside IRB Approval

Attach the IRB Approval from the Outside IRB.

Outside IRB Review Meeting Minutes

Attach the minutes from the outside IRB meeting(s) for the review of this study.

Outside IRB Correspondence

Attach all correspondence concerning the review of this study by the Outside IRB.

Modification Submission

Modification

IMPORTANT REMINDER

Any changes to the study protocol **must** be included in a modification submission, including but not limited to:

- Any changes to the **target subject population**, including but not limited to age, race, disability status, and gender
- Any changes to previously approved study procedures
- Any changes to request participant totals
- Any change to investigators or research personnel

*required

Are you making changes to the project?

✓ Yes

Please make your changes in the sections to the left.

No

*required

Justification

Please provide the reasons for the modifications.

We are adding additional personnels to this project.

Weber State University IRB

Welcome and Thank You for your interest in completing human subjects research at or in association with Weber State University!

Please keep the following in mind as you complete your application:

- Incomplete submissions will not be reviewed.
- You cannot begin data collection until a formal approval letter from the IRB has been received.
- The IRB meets as needed during the academic year. Please submit the application as soon as possible and allow at least 7 days for an expedited review and 30 days for a Full Board Review.

About Cayuse IRB Software

Cayuse IRB is an interactive web application. As you answer questions, new sections relevant to the type of research being conducted will appear on the left-hand side. Therefore not all numbered sections may appear. You do not have to finish the application in one sitting. All information can be saved.

Additional information has been added throughout the form for guidance and clarity. That additional information can be found by clicking the question mark it the top-right corner of each section.

For more information about the IRB submission Process, IRB Tracking, and Cayuse IRB Tasks, please refer to the Cayuse IRB Procedures Manual.

Getting Started

Throughout the submission, you will be asked to provide the following (as applicable):

- Detailed Study Information
- Informed Consent Forms (in appropriate languages)
- Study Recruitment Documents (flyers, social media posts, recruitment scripts, etc)
- Copies of CITI training certificates for all study personnel
- All instrument, questionnaires, surveys, interview questions, discussion questions
- Letters of support from any sponsoring or supporting institutions/organizations
- Photographic/Video Release from research participants.

*required

IRB Subcomittee

Please indicate the most appropriate Weber State IRB Subcommittee to review your research project.

NOTE: In nearly all instances this is the College the PI is employed.
CCEL, the Library and other campus entities existing outside of a College should select AD Hoc Review.

College of Arts & Humanities

School of Business & Economics

✓ College of Education

College of Engineering, Applied Science & Technology

College of Health Professions

College of Science

College of Social and Behavioral Sciences

College of Social and Behavioral Sciences - Psychology

Ad Hoc Review

*required

Researcher Agreement

This research study involves the use of human subjects. I understand the university's policy concerning research involving human subjects and by submitting this application I agree to:

- Obtain voluntary and informed consent of subjects who are to participate in this project.
- Report to the IRB any unanticipated effects on subjects which become apparent during the course of, or as result of, the experimentation and the actions taken.
- Cooperate with members of the committee charged with continuing review of this project.
- Obtain prior approval from the committee before amending or altering the scope of the project or implementing changes in the approved consent document.
- Maintain the documentation of consent forms and progress reports as required by institutional policy for three years.
- Safeguard the confidentiality of research subjects and the data collected when the approved level of research requires it.

I have read the information above and I am ready to begin my submission.

✓ Yes

2- Submission Information

*required

What type of activity is this submission for?

- ✓ Research Study
- Quality or Process Improvement Project - Medical or Health Related
- Quality or Process Improvement Project - NON Medical or Health Related
- Clinical Trial
- Single Patient, Treatment Use, Continued Access Drug/Device Study
- Emergency (or Compassionate) Use of Investigational Drug or Device
- Student Directed Classroom-Based Research

*required

Is this a multi-institutional study?

- Yes
- ✓ No

*required

Research Categorization

Do you believe the research in this application meets the criteria for exemption?

- Yes
- ✓ No

*required

Select the level of review you believe is appropriate for this research

---

✓ Expedited - low to moderate risk

Full Board - moderate to high risk; research involving protected status groups or individuals

3- Study Information

*required

What is your status at Weber State University?

✓ Faculty

Student

Staff

Other

Study Personnel

Note: If you cannot find a person in the people finder, please contact the IRB Office.

*required

Principal Investigator

*Provide the name of the Principal Investigator of this study. This individual is in charge of the research team and must be a Weber State employee (Faculty or Staff). Students may **NOT** serve as a sole PI, Graduate students may serve as a CO-PI, undergraduate students may be on the research team but may **NOT** serve as a PI.*

Name: Saori Hanaki
Organization: Exercise and Nutrition Science
Address: 1435 Village Dr DEPT 2805 , Ogden, UT 84408-2805
Phone: 8016266626
Email: saorihanaki@weber.edu

Co-Principal Investigator(s)

Provide the name(s) of Investigator(s) for this study.

Name: Stacie Wing-Gaia
Organization: Exercise and Nutrition Science
Address: 1435 Village Dr DEPT 2805 , Ogden, UT 84408-2805
Phone: 8016268942
Email: swinggaia@weber.edu

Name: Damon Joyner
Organization: Exercise and Nutrition Science
Address: 1435 Village Dr DEPT 2805 , Ogden, UT 84408-2805
Phone: 8016266627
Email: damonjoyner@weber.edu

Name: Bryan Dowdell
Organization: College of Education
Address:
Phone:
Email: bryandowdell@weber.edu

Name: David Aguilar-Alvarez
Organization: Exercise and Nutrition Science
Address: 1435 Village Dr DEPT 2805 , Ogden, UT 84408-2805
Phone: 8016268867
Email: daguilaralvarez@weber.edu

*required

Primary Contact

Provide the name of the Primary Contact of this study. This person does **NOT** have to be the PI and should be the person responsible for corresponding with the IRB and the investigative team.

Name: Bryan Dowdell
Organization: College of Education
Address:
Phone:
Email: bryandowdell@weber.edu

Other Personnel - WSU Affiliated

---

*Provide the name(s) of **ALL** other personnel for this study. Anyone who will have direct contact with participants and/or access to identifiable participant data.*

Other Personnel - NON WSU Affiliated

---

*Provide the name(s) of **ALL** other personnel **NOT AFFILIATED** with WSU (individuals without WSU log in credentials) for this study. Anyone who will have direct contact with participants and/or access to identifiable participant data.*

-Please provide Name, Affiliation and Email address.

New personnel added are in BOLD

The following staff/faculty at Weber State will also be co-investigators (their names are not in this system for whatever reason):

- Qi Jin, assistant professor in Exercise and Nutrition Sciences (ENS), qijin1@weber.edu
- Jamie Stein, instructor in ENS, jamiestein@weber.edu

- Sara Phlypo, ENS Lab coordinator, saraphlypo1@weber.edu

Following WSU undergraduate students will be assisting this project for undergraduate-level research experience:

- **Lindsey Johnson, undergraduate student, lindsayjohnson2@mail.weber.edu**
- **Chandler Williams, undergraduate student, chandlerwilliams@mail.weber.edu**
- **Alonna Jones, undergraduate/future graduate student, alonnajones@mail.weber.edu**

Following are the recent WSU graduates of Spring 2024 who will be assisting this project:

- **Mason Masters, masonmasters@mail.weber.edu**

The following non-WSU affiliated individual will also be assisting with data collection and analysis for graduate-level research experience:

- Anthony Ludwig, graduate student at SJSU, anthony.f.ludwig@gmail.com
- **Jim Healis, former WSU undergraduate student and former SUS graduate student, no current affiliation, jhealis@gmail.com**

*required

Study Site

Please select all sites at which research will be conducted.

✔ Weber State University

At which Weber State University facilities will this research take place. (select all that apply)

Main Campus - Ogden

West Center

Farmington Station

Community Education Center - Ogden

Center for Continuing Education - Clearfield

Davis

Morgan Center

Other WSU affiliated site(s)

✓ External Site (non Weber State University)

Please provide the names of the external collaborating sites.

Some data collection will be conducted in our reserved sites near the finish line and vendors of the Ogden Marathon Expo in downtown Ogden. Blood and urine samples will be stored/analyzed in the Nutrition biochemistry lab in Swenson Hall.

Study Dates

Please provide the PROJECTED study start and end dates. Projects may not begin until approval letter is received.

*required

Start Date

01-01-2024

*required

End Date

12-31-2024

Departmental Approval

*required

Does your Department require approval of IRB submissions?

✓ Yes

*required

Please upload a signed departmental approval form.

Download Form

Departmental Form IRB Ogden Marathon.pdf

No

Subject Enrollment

Enter the number of subjects that will be enrolled in this study.

*required

Total Study Enrollment

Please enter the total number of subjects to be enrolled at all study sites.

50. We are enrolling 20 subjects for the full protocol. We would like to recruit 30 more subjects for a partial protocol (these subjects would only complete surveys/questionnaires).

*required

Enrollment at Weber State University

*Please enter the number of subjects that will be enrolled at **Weber State University**.*

0-50

*required

Ages

Select the age range of subjects that will be enrolled in this study. Check all that apply.

Fetus

Birth to less than 1 month

1 month to less than 12 years old

12 years old and less than 18 years old

✓ 18 years and older

*required

Vulnerable Populations

Please check the population(s) that will be ***TARGETED*** *for enrolled. Check all that apply.*

Fetuses

Pregnant Women

Minors with Parental Consent

Minors who can Consent Themselves (emancipated minors, minors in states that allow consent; please direct any questions about this to the IRB office)

Prisoners

Individuals with Cognitive Impairments

Economically Disadvantaged Persons

Educationally Disadvantaged Persons

Other

✓ None of the Above

*required

Research Summary and Justification

Please provide a detailed summary that includes background and justification for the proposed research.

Please limit this section to 1000 words, citations should be included as a separate Appendix if necessary.

This research proposal is inherently an extension of our department's (Exercise and Nutrition Sciences; ENS) recently awarded Ambrose Amos Shaw Endowed Chair Award. This was awarded to promote a collaborative research opportunity for faculty and students alike to investigate characteristics and biomarkers in marathon runners.

Marathon running induces considerable physiological, psychological, and biomechanical strain on participants (Sanchez, Corwell, & Berkoff, 2006). The duration of exercise required to complete a marathon increases biological markers of cardiac stress (e.g. Troponin T), inflammation (e.g. TNF-α), and intestinal permeability (e.g. Fatty Acid Binding Protein) (Bernat-Adell et al., 2019; Da Ponte et al., 2018; Karhu et al., 2017; Scherr et al., 2011). Further, nutritional practices leading up to and during a marathon have considerable impacts on performance and recovery (Almond et al., 2005; Mielgo-Ayuso et al., 2020). The repetitive eccentric contractions occurring during running combined with the long distance (26.2 miles), provides the perfect scenario to induce considerable muscle damage (Clarkson, 2007; Del Coso et al., 2013; Kyröläinen et al., 2000). As such, many biological and biomechanical markers representing this damage are elevated following a marathon (Del Coso et al., 2013; Mielgo-Ayuso et al., 2020). This may have impacts on many biomechanical properties that affect gait, stride length, stride rate, running economy, and ultimately function (Del Coso et al., 2013; Kyröläinen et al., 2000). Muscle damage not only leads to biomechanical changes and soreness, but also challenges other physiological systems to maintain function. For example, certain cellular contents released from damaged muscle fibers (e.g. myoglobin) into the circulation can have nephrotoxic effects (i.e. damage the kidneys), which may compromise overall renal function (Bosch, Poch, & Grau, 2009; Clarkson, 2007). When running a marathon, the renal system is stressed due to reduced perfusion associated with exercise and is often combined with hypovolemia from dehydration (Mansour et al., 2017; Sanchez et al., 2006). Therefore, adding a toxic substance such as myoglobin, damages the nephrons and leads to elevations in biological markers of acute kidney injury post-race (e.g. creatinine, kidney injury molecule-1) (Mansour et al., 2017; McCullough et al., 2011; Mingels, Jacobs, Kleijnen, Wodzig, & Dieijen-Visser, 2009). The responses to marathon running are variable in the literature, due to differences in weather, altitude, and course design. This variation is beneficial for providing a wide response pattern and allows for a personalized assessment for expected results given specific conditions.

As such, the purpose of this study is to evaluate nutritional behaviors and the biomechanical and physiological responses to running the Ogden Marathon. This marathon course contains a considerable downhill component, which facilitates muscle damage more-so than flat marathons. Therefore, we expect considerable physiological and biomechanical strain that may lead to delayed recovery. Further, we intend to assess variables pre-marathon, immediately post-marathon, and at 48 hours post-marathon. Unfortunately, relatively few studies have evaluated the effects of completing a marathon past 24 hours, likely due to participant availability. The relative lack of data for responses past 24 hours is concerning as

many biological markers do not peak until as late as 72 hours (e.g. creatine kinase) or may have variations that peak immediately and then again after several days (e.g. kidney injury molecule 1). Thus, we propose to investigate these variables at various time points to contribute to a needed area in research.

Appendix

Please attach appendix document(s) as needed.

Appendix A - References.docx

*required

Hypothesis

Provide the study hypothesis.

We hypothesize that variables associated with muscle damage will remain elevated at the 48-hour follow-up, however only certain physiological variables (e.g. kidney injury molecule-1) will be increased following recovery.

*required

Objectives

Provide the study objectives.

The Ogden Marathon is unique in that its course has a considerable downhill component. This uniqueness allows us to analyze and contribute to the growing body of variable response patterns for various marathons.

- Assess baseline biomarkers as well as up to 48 hours post-marathon for analysis.
- Analyze health patterns, biomechanics, and nutrition habits to further explore potential covariates and relationships.

*required

Outcome Measures

Provide the main study outcome measures/dependent variables.

We will mainly be assessing muscle damage biomarkers and hydration markers via blood and urine samples at various time points. We will also be collecting the following outcome measures to assess further relationships:

- Scales for thirst perception (detailing the level or sensation of thirst), muscle pain (how much pain they are feeling), and fatigue (how tired they feel) to evaluate the perceptual strain.
- Body weight and composition before and after the race
- Skin carotenoid levels before and after race
- Training/medical history
- Questionnaires detailing participant NSAID use will be completed before, following, and after 48 hours of recovery.
- Dietary patterns such as caloric intake and nutrient breakdown
- Run/stride frequency during race
- Isometric strength before and after race

*required

Inclusion Criteria

List and describe the inclusion criteria.

- Participants to be between the ages of 18–65 years old
- Participants are already registered and running the full Ogden Marathon

*required

Exclusion Criteria

List and describe the exclusion criteria.

Exclusion Criteria is only for the full protocol:

- No known cardiovascular, respiratory, gastrointestinal bleeding, inflammatory, metabolic/ fluid-electrolyte disorder or other chronic diseases, or pregnant
- Prone to vasovagal syncope as a result of venipuncture
- Not able to attend all visits

6- Study Procedures

*required

Describe all study procedures.

Provide a complete and thorough description of the procedures in the proposed research.

This description should encompass the experimental course of a subject from their entry into the study to their completion of the study.

This study's protocol is multi-faceted. We will recruit subjects through Ogden's GOAL (Get Out And Live) Foundation whom we have already been approved and partnered with for race day logistics (see attached MOU in the Appendix). This study will consist of a familiarization and three visits (pre-race expo, race day, and 48-hour follow-up). The familiarization (in-person or virtual Zoom) will include completing the informed consent form, followed by dietary surveys (Appendix) and a 24 h history questionnaire (Appendix) The participant will also be enrolled in a Strava group with de-identified codes. Following completion of thes forms, demographic information (e.g. height, weight, age, gender, and ethnicity) will be collected. Meeting times will then be scheduled for the day before the race (at the expo) and the morning of the race.

During visit one at the expo, participants will also be instructed to record dietary intake for prior to the race, during the race day, and following the race using the automated self-administered 24 hr recall (ASA24-202 Appendix). The participants will be provided a login specific to their participant ID, but deidentified to protec their identity. Participants will also complete a training/health history survey and body weight/composition assessment. Lastly, they will be given a urine sample collection kit and further instructions, checks, and reminders for race day.

On the day of the race (visit two), the participant will report to the designated area near the shuttle location that will transport them to the race start line. Upon arrival, participants will be asked to provide the urine sample in a urine specimen cup and have their body mass measured on a standard research body mass scale. Hydration will be analyzed using urine specific gravity via refractometer, urine color, and urine osmolality via freezing point depression (Advanced Instruments osmometer). Urine will also be stored for la analysis of biological markers (i.e. inflammatory markers, renal biomarkers, cytokines, immune markers, markers of metabolism, cardiovascular markers). Participants will place their hand on our carotenoid senso to measure skin carotenoid levels. Participants will also complete a pre-race questionnaire (Appendix), pair fatigue, and thirst scales (Appendix) as well as record body weight/body composition through our InBody scale. They will also be tested for isometric leg strength on both legs with a portable force transducer. A sm subset of runners (n=10) will also be instrumented with a RunScribe device on their shoe that will track biomechanical information. A small blood sample (~20 mL or ~1.4 tablespoon/one tube) will be obtained via venipuncture from the antecubital vein from trained phlebotomists. Blood samples will be used to analyze osmolality and saved for later analysis of biological markers (i.e. inflammatory markers, renal biomarkers, cytokines, immune markers, markers of metabolism, cardiovascular markers). Participants will then be provided a wristband or stamp that will indicate their participation for identification after the race by researchers. They will then go complete the race.

Upon finishing the race, the participants will be asked to report to the data collection tent near the finish line Upon reporting the participant will provide a urine sample, body mass/composition measurement, and bloo sample via venipuncture from the antecubital vein (~20 ml or one tube). Participants will also complete a post-race questionnaire (Appendix), pain, fatigue, and thirst scales, and provide a description of their dietar intake during the race. They will also be re-tested for isometric leg strength on both legs. The runners wear

the RunScribe units will also be de-instrumented. Participants will then be provided beverages and snacks desired) and they will schedule a time to come to their 48-hour follow-up visit at the Weber State University Human Performance Laboratory. Participants will continue to record dietary intake using the ASA24-2022 f an additional 2-days post-race.

Upon arriving for the 48-hour follow-up visit (visit three), participants will provide a urine sample, body mass measurement, and blood sample via venipuncture from the antecubital vein (~20 ml or two tubes). Participants will also complete a recovery questionnaire (Appendix J), pain, fatigue, and thirst scales. They will also be tested for isometric leg strength on both legs. Participants will also be instructed to change into tight fitting clothing (e.g. spandex shorts, sports bra, bathing suit) to complete a BodPod assessment of boc composition. Once complete the participants will be provided a copy of their BodPod results.

For participants who elect to partake in the partial protocol, they will only have to complete the familiarizatic and complete the online ASA24 dietary recalls on the same days as the full protocol.

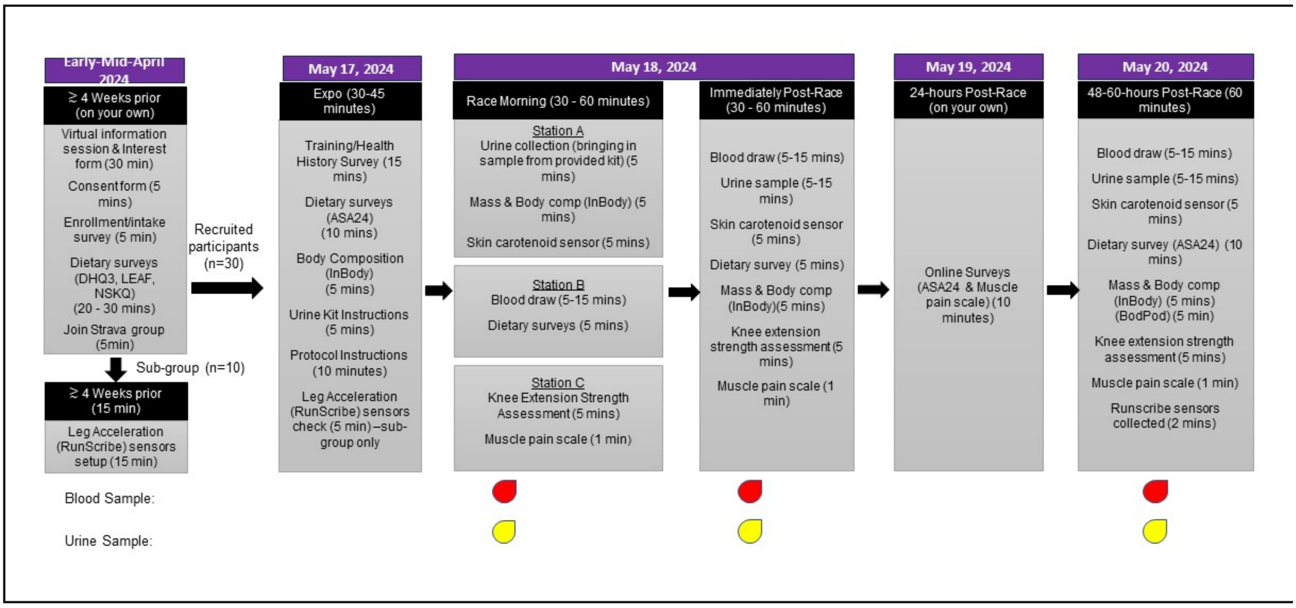

*required

Describe your subject recruitment procedures and any material inducements given for participation

Recruitment will occur via word of mouth, emails sent to race participants, and social media postings such as Facebook pages. Recruitment statements will read as follows: Participants needed for study runners completing Ogden Marathon.

- Participants (up to 20) for the full research protocol including recording your diet and providing urine and blood samples can receive a VIP shuttle to the marathon starting line, report of body composition via BodPod, and $100 gift card after completing the study.
- Additional participants are sought to completes surveys only (no urine and blood samples). The survey-only participants are not eligible for the gift card or a body composition analysis.

In order to participate, you must be already registered for and run the Ogden Full Marathon and not pregnant. If interested, please visit to this link (link to an online interest form). If you have any questions, contact Dr. Saori Hanaki at saorihanaki@weber.edu, Dr. Stacie Wing-Gaia at swinggaia@weber.edu, Dr. Bryan Dowdell at bryandowdell@weber.edu, Dr. Damon Joyner at damonjoyner@weber.edu, Dr. Qi Jin at qijin1@weber.edu, or Dr. David Aguilar Alvarez at daguilaralvarez@weber.edu.

*required

Describe the duration of study participation, the length and number of study visits, and the timetable for study completion.

Please be sure to provide the following:

- How many times human subjects will perform research activities;
- How long each visit will take;
- Total time requirement for a subject to complete the proposed research?

Participants will complete a familiarization visit and three additional visits (pre-race expo, race day, and 48-hour recovery day). The familiarization should take ~30 minutes. The pre, post, and 48 hour recovery visits will take ~30 minutes. The surveys completed on their own take ~15 minutes each. Therefore total time commitment is ~3 hours.

*required

Describe the information to be gathered and the means for collecting and recording data.

Many studies use multiple instruments/questionnaires/surveys as part of the research methodology. If applicable to the proposed research, list each instrument/questionnaire/survey that will be administered to subjects and provide a rationale for the inclusion of each one.

If previously collected data is to be used, describe both the previous and proposed uses of these data.

- Hydration will be assessed via a refractometer, urine color chart, and osmometer from urine samples.
- Blood biomarkers associated with muscle damage will be assessed via assay kits.
- Scales for thirst perception (detailing the level or sensation of thirst), muscle pain (how much pain they are feeling), and fatigue (how tired they feel) to evaluate the perceptual strain.
- Body weight and composition via Inbody scale and Bod Pod.
- Skin carotenoid levels via skin carotenoid sensor.
- A training/medical history questionnaire will be used to evaluate health history and quantify the level of activity in which individuals regularly engage.
- Questionnaires detailing participant NSAID use will be completed before, following, and after 48 hours of recovery.
- A validated online diet recording survey (ASA24) as well as DHQ3, LEAF, NSKQ will be used to quantify macronutrient and micronutrient intake for the duration of the study.
- RunScribe sensors will be attached to select participants' shoes to identify their biomechanical responses.
- Isometric strength will be assessed using a force transducer to identify changes associated with muscle damage.

Study Instruments

Attach all instruments (i.e. personality scales, questionnaires, evaluation blanks, etc) to be used in the study.

ACSM Health History & Physical Activity Questionnaire.pdf

Nutrition Knowledge Survey.docx

McKay Training Status Classification.pdf

The LEAF-Questionnaire.pdf

Sample questionnaire DHQ3.pdf

ASA24 Info.docx

Ogden Marathon Data Collection Checklist.docx

*required

Survey, Questionnaire, or Interview

Will the study utilize surveys, questionnaires, or interviews?

✓ Yes

*required

Attach all copies of surveys, questionnaires, or interviews.

If your survery is hosted on an online application, WSU IRB prefers a link to the study. Please use the attached link function.

ACSM Health History & Physical Activity Questionnaire.pdf

Nutrition Knowledge Survey.docx

McKay Training Status Classification.pdf

The LEAF-Questionnaire.pdf

Sample questionnaire DHQ3.pdf

ASA24 Info.docx

Ogden Marathon Data Collection Checklist.docx

No

*required

Will the survey, questionnaire, or interview record any information that can identify the participants?

Yes

✓ No

*required

Genetic Testing

Will this study involve genetic testing?

Yes

✓ No

*required

Drugs, Devices, Biologics

Will the study involve administering any of the following? Check all that apply.

Drug

Biologic

✓ Device

*required

Please describe.

A RunScribe motion sensor device will be placed on a subset of the subject's shoes during the race.

None of the above

*required

Participant Data, Specimens, and Records

Does this project involve the collection or use of materials (data or specimens) recorded in a manner that could identify the individuals who provided the materials, either directly or through identifiers linked to these individuals?

Yes

✓ No

*required

Specimen and Sample Retention

Does the research team plan to retain any Specimens or Biologic samples for future analysis.

✓ Yes

*required

Please explain the intent of the retention and expected destruction date.

NOTE: Intend to retain samples for future analysis **MUST** be clearly stated in your informed consent document and disclosed to participants.

All blood and urine samples will be properly stored in our biochemistry lab (SW 133) for future biomarker analysis. These samples will be retained for the maximal allowable time of three years after which all samples will be properly disposed of according to university and OSHA protocols.

No

7- Participant Protection

Risk: *Generally, risk assessment in research considers the harm, trauma, discomfort, stress, or any other undesirable or untoward consequence of being a research subject whether anticipated or unexpected. Risk may take the following forms: physical, psychological, emotional, financial, and/or social. This list is representative but not comprehensive.*

Please consider the following questions based on the experiences that subjects might encounter through participation in the proposed research.

---

*required

Potential Risk

Whether great or small, do you think there is potential risks to subjects that participate in the proposed research.

✓ Yes

No

Potential Risks

---

*required

Describe immediate risks, long-term risks, rationale for the necessity of such risks, alternatives that were or will be considered, and why alternatives may not be feasible.

There are multiple risks associated with this study. Most of these risks are inherent and cannot be avoided because of the purpose of the study, thus alternatives are not feasible.

The full protocol (includes blood/urine samples) risks are:

- Inherent risks associated with voluntary exercise and exertion
- Inherent risks associated with blood draws such as feelings of discomfort and pain associated with needles as well as vasovagal syncope (fainting upon sight of blood/needles)

- Subjects are required to answer questions from health surveys or questionnaires such as dietary recalls that may induce feelings of discomfort.
- We cannot guarantee absolute confidentiality

The partial protocol (only includes surveys/questionnaires) risks are:

- Subjects are required to answer questions from health surveys or questionnaires such as dietary recalls that may induce feelings of discomfort.
- We cannot guarantee absolute confidentiality

What steps will be taken by the investigator to reduce the aforementioned risks?

- Exclusion criteria of pre-existing conditions to mitigate inherent risks associated with exercise (anybody with CVD diseases, pulmonary conditions, etc cannot participate).
- Exclusion criteria of those weary of blood draws (i.e. prone to vasovagal responses such as fainting upon the sight of blood/needles during blood draws).
- We will be using trained phlebotomists for the blood draws. Universal precautions (PPE) will be utilized to minimize any chance of infection. The participants will be in a seated position during the blood draw (minimizing any injury should they feel lightheaded). Also, pressure will be applied to the blood draw sight to minimize soreness.
- Subjects will be reminded that this study is entirely voluntary. If they want to discontinue participating, they may drop out at any time and contact research personnel.
- All surveys and assessments will be completed by a trained research staff member.
- All data will be de-identified.

*required

Describe any potential legal, financial, social, or personal affects on subjects.

None

What steps will be taken by the investigator to reduce the aforementioned risks?

---

*required

Will deception be used as a method of data gathering?

Yes

✓ No

*required

Does this research require face to face contact between researchers and participants

---

✓ Yes

*required

Please detail your COVID 19 Mitigation Procedures.

If required by the university, the IRB, and/or the state of Utah, all subjects and researchers will wear masks (face coverings) at all times.

No

*required

Expected Benefits

Please describe any benefits that research subjects will receive as a direct result of their participation in the proposed research.

Note: *compensation is not considered a benefit*

Subjects will be given interesting information about their heath habits and body composition through assessments that normally cost money. In addition, subjects are given access to complimentary and private access to a bus shuttle and restrooms before and after the marathon designated only for study subjects.

Please describe how this research may provide benefit to scientific knowledge, a specific discipline, and/or society in general.

By participating and contributing to data collection, subjects can know that the findings from this research will contribute to a missing area of research in terms of marathon responses.

*required

Subject Compensation

*required

Will subjects receive any form of compensation for participation in this investigation?

Compensation includes but is not limited to: University Credit, extra points in a course, gift cards or any form of monetary instrument.

✓ Yes

No

*required

Please describe and detail any and all forms of compensation that subjects may receive.

$100 gift card

*required

Safeguarding Subjects' Identity

What uses will be made of identifiable information obtained from the subjects?

The only identifiable information (name) will be the signed informed consent form. Signed informed consent forms will be kept separated from data collected, and the signed/completed hard copy documents will be stored in a locked cabinet in our (ENS) lab coordinator's office (Sara Phlypo who is part of research personnel).

What precautions will be taken to safeguard identifiable records or individuals?

The only identifiable information (name) will be the signed informed consent form. Signed informed consent forms will be kept separated from data collected, and the signed/completed hard copy documents will be stored in a locked cabinet in our (ENS) lab coordinator's office (Sara Phlypo who is part of research personnel).

*required

Data Management

Protecting ***hard copy*** data may involve de-identification of data (see:NISTIR-8053) and secured storage locations and conditions (see: Weber State University PPM 10-1).

Describe what type of hard copy data will be generated by the proposed research (i.e.,

notes, audio/video tapes, questionnaires, etc.). Where will this hard copy data be stored and how will it be protected?

The only identifiable information (name) will be the signed informed consent form. Signed informed consent forms will be kept separated from data collected, and the signed/completed hard copy documents will be stored in a locked cabinet in our (ENS) lab coordinator's office (Sara Phlypo who is part of research personnel).

Hard copy surveys/questionnaires (such as the nutritional and dietary surveys) and the stored biologicals samples (in SW 133 biochemistry lab) will use de-identified codes (i.e. OMS-01). Hard copy data will be stored securely and separately from the informed consents in our lab coordinator's office. Biological samples will be stored securely in our -80 degree freezer in SW 133 biochemistry lab in which only authorized users have access.

Protecting ***electronic data*** may involve a secure network, password access, and data de-identification/ encryption (see: Weber State University PPM 10-1)

Describe what type of electronic data will be generated by the proposed research (i.e., computer files/ spreadsheets, questionnaires, images, video, audio/mp3 files, etc.) Where will this electronic data be stored and how will it be protected

All questionnaire results (de-identified) will be entered into data spreadsheets saved in a Box/Google Drive Folder which only the study investigators will have access to. These are password-protected and managed by the university. The ASA24 platform data are stored under a subject's ID number without any identifiable information. ASA24 platforms are password-protected and only the research personnel will have access to it. The exported data from this platform will be stored in the Google Drive/Box mentioned above.

*required

Will the research require accessing student educational records?

Yes

✓ No

*required

On what date will study data and materials with identifiable information be destroyed?

Please select a year and date.

12-31-2027

*required

Will raw data be made available to anyone other than the principal investigator and the immediate study personnel?

Yes

✓ No

*required

Informed Consent

Describe the procedures for obtaining informed consent.

Informed Consent will be obtained in either hard-copy form or virtually via a live Zoom session with recruited participants. We will have two separate Informed Consent forms: one for those interested in completing the full protocol (which includes blood draws and urine samples) and one for the partial protocol (which only includes the survey/questionnaires).

*required

Please attach the your Informed Consent Document(s). Please include all versions/languages you intend to utilize. **PDF format required!**

You will also have a chance to attach on the last page of the application.
Download Template (Word Doc Format)
Informed Consent Full Protocol.doc

Informed Consent Partial Protocol.docx

8- Conflict of Interest

*required

Do you or any investigator(s) participating in this study have a financial interest related to this research project?

Yes

✓ No

Study Funding

*required

Does this study have **EXTERNAL** funding

Yes

✓ No

*required

Does this study have an **INTERNAL** funding sources

✓ Yes

Please list sources of INTERNAL funding.

Ambrose Amos Shaw Endowed Chair Award

No

9- Attachments

Participant Protection

Informed Consent Form

Please upload **ALL** versions of the Informed Consent document that maybe used. If requesting a waiver please upload justification here. **PDF format required!**
Informed Consent Full Protocol.doc

Informed Consent Partial Protocol.docx

If requesting an Informed Consent Waiver/Alternation under 45 CFR 46.16 please explain.

*required

Research Human Subjects Training Certificate(s)

Please upload a CITI certificate for **EVERY** investigator listed on the application
Citi Certificate - Jamie Stein.pdf

CITI certificate Damon Joyner.pdf

CITI DavidAguilar.pdf

CITI_qijin.pdf

citiCompletionCertificate_Dowdell_2023.pdf

Stacie Wing-Gaia CITI Completion certificate.pdf

citiCompletionReportHanakiJan2021.pdf

citiCompletionCertificate_Sara Phlypo.pdf

citiCompletionCertificate_Lindsay_Johnson.pdf

citiCompletionCertificate_Mason_Masters.pdf

CITI Program Completion Certificate Chandler_Williams.pdf

citiCompletionCertificate_Jim_Healis.pdf

CITI completion certificate Alonna Jones.pdf

Study Procedures

Study Documents

If applicable, this includes flyers used for recruitment.
Recruitment announcement.docx

Study Instruments

Attach all instruments (i.e. personality scales, questionnaires, evaluation blanks, etc) to be used in the study.
ACSM Health History & Physical Activity Questionnaire.pdf

Nutrition Knowledge Survey.docx

McKay Training Status Classification.pdf

The LEAF-Questionnaire.pdf

Sample questionnaire DHQ3.pdf

ASA24 Info.docx

Ogden Marathon Data Collection Checklist.docx

FDA Letter

Study Design

Appendix

Appendix A - References.docx

External IRB of Record

Study Protocol

Attach the protocol for this study that was reviewed by the Outside IRB.

Outside IRB Approval

Attach the IRB Approval from the Outside IRB.

Outside IRB Review Meeting Minutes

Attach the minutes from the outside IRB meeting(s) for the review of this study.

Outside IRB Correspondence

Attach all correspondence concerning the review of this study by the Outside IRB.

Modification Submission

Modification

IMPORTANT REMINDER

Any changes to the study protocol **must** be included in a modification submission, including but not limited to:

- Any changes to the **target subject population**, including but not limited to age, race, disability status, and gender
- Any changes to previously approved study procedures
- Any changes to request participant totals
- Any change to investigators or research personnel

*required

Are you making changes to the project?

✓ Yes

Please make your changes in the sections to the left.

No

*required

Justification

Please provide the reasons for the modifications.

Adding a student with CITI training to the IRB so that they may disseminate data.

1- Getting Started

Weber State University IRB

Welcome and Thank You for your interest in completing human subjects research at or in association with Weber State University!

Please keep the following in mind as you complete your application:

- Incomplete submissions will not be reviewed.
- You cannot begin data collection until a formal approval letter from the IRB has been received.
- The IRB meets as needed during the academic year. Please submit the application as soon as possible and allow at least 7 days for an expedited review and 30 days for a Full Board Review.

About Cayuse IRB Software

Cayuse IRB is an interactive web application. As you answer questions, new sections relevant to the type of research being conducted will appear on the left-hand side. Therefore not all numbered sections may appear. You do not have to finish the application in one sitting. All information can be saved.

Additional information has been added throughout the form for guidance and clarity. That additional information can be found by clicking the question mark it the top-right corner of each section.

For more information about the IRB submission Process, IRB Tracking, and Cayuse IRB Tasks, please refer to the Cayuse IRB Procedures Manual.

Getting Started

Throughout the submission, you will be asked to provide the following (as applicable):

- Detailed Study Information
- Informed Consent Forms (in appropriate languages)
- Study Recruitment Documents (flyers, social media posts, recruitment scripts, etc)
- Copies of CITI training certificates for all study personnel
- All instrument, questionnaires, surveys, interview questions, discussion questions
- Letters of support from any sponsoring or supporting institutions/organizations
- Photographic/Video Release from research participants.

*required

IRB Subcomittee

Please indicate the most appropriate Weber State IRB Subcommittee to review your research project.

NOTE: In nearly all instances this is the College the PI is employed.
CCEL, the Library and other campus entities existing outside of a College should select AD Hoc Review.

College of Arts & Humanities

School of Business & Economics

✓ College of Education

College of Engineering, Applied Science & Technology

College of Health Professions

College of Science

College of Social and Behavioral Sciences

College of Social and Behavioral Sciences - Psychology

Ad Hoc Review

*required

Researcher Agreement

This research study involves the use of human subjects. I understand the university's policy concerning research involving human subjects and by submitting this application I agree to:

- Obtain voluntary and informed consent of subjects who are to participate in this project.
- Report to the IRB any unanticipated effects on subjects which become apparent during the course of, or as result of, the experimentation and the actions taken.
- Cooperate with members of the committee charged with continuing review of this project.
- Obtain prior approval from the committee before amending or altering the scope of the project or implementing changes in the approved consent document.
- Maintain the documentation of consent forms and progress reports as required by institutional policy for three years.
- Safeguard the confidentiality of research subjects and the data collected when the approved level of research requires it.

I have read the information above and I am ready to begin my submission.

✓ Yes

2- Submission Information

*required

What type of activity is this submission for?

✓ Research Study

Quality or Process Improvement Project - Medical or Health Related

Quality or Process Improvement Project - NON Medical or Health Related

Clinical Trial

Single Patient, Treatment Use, Continued Access Drug/Device Study

Emergency (or Compassionate) Use of Investigational Drug or Device

Student Directed Classroom-Based Research

*required

Is this a multi-institutional study?

Yes

✓ No

*required

Research Categorization

Do you believe the research in this application meets the criteria for exemption?

Yes

✓ No

*required

Select the level of review you believe is appropriate for this research

✓ Expedited - low to moderate risk

Full Board - moderate to high risk; research involving protected status groups or individuals

3- Study Information

*required

What is your status at Weber State University?

✓ Faculty

Student

Staff

Other

Study Personnel

Note: If you cannot find a person in the people finder, please contact the IRB Office.

*required

Principal Investigator

*Provide the name of the Principal Investigator of this study. This individual is in charge of the research team and must be a Weber State employee (Faculty or Staff). Students may **NOT** serve as a sole PI, Graduate students may serve as a CO-PI, undergraduate students may be on the research team but may **NOT** serve as a PI.*

Name: Saori Hanaki
Organization: Exercise and Nutrition Science
Address: 1435 Village Dr DEPT 2805 , Ogden, UT 84408-2805
Phone: 8016266626
Email: saorihanaki@weber.edu

Co-Principal Investigator(s)

Provide the name(s) of Investigator(s) for this study.

Name: Stacie Wing-Gaia
Organization: Exercise and Nutrition Science
Address: 1435 Village Dr DEPT 2805 , Ogden, UT 84408-2805
Phone: 8016268942
Email: swinggaia@weber.edu

Name: Damon Joyner
Organization: Exercise and Nutrition Science
Address: 1435 Village Dr DEPT 2805 , Ogden, UT 84408-2805
Phone: 8016266627
Email: damonjoyner@weber.edu

Name: Bryan Dowdell
Organization: Exercise and Nutrition Science
Address:
Phone:
Email: bryandowdell@weber.edu

Name: David Aguilar-Alvarez
Organization: Exercise and Nutrition Science
Address: 1435 Village Dr DEPT 2805 , Ogden, UT 84408-2805
Phone: 8016268867
Email: daguilaralvarez@weber.edu

*required

Primary Contact

Provide the name of the Primary Contact of this study. This person does **NOT** have to be the PI and should be the person responsible for corresponding with the IRB and the investigative team.
Name: Bryan Dowdell
Organization: Exercise and Nutrition Science
Address:
Phone:
Email: bryandowdell@weber.edu

Other Personnel - WSU Affiliated

Provide the name(s) of ***ALL*** *other personnel for this study. Anyone who will have direct contact with participants and/or access to identifiable participant data.*

Other Personnel - NON WSU Affiliated

Provide the name(s) of ***ALL*** *other personnel* ***NOT AFFILIATED*** *with WSU (individuals without WSU log in credentials) for this study. Anyone who will have direct contact with participants and/or access to identifiable participant data.*

-Please provide Name, Affiliation and Email address.

New personnel added are in BOLD

The following staff/faculty at Weber State will also be co-investigators (their names are not in this system for whatever reason):

- Qi Jin, assistant professor in Exercise and Nutrition Sciences (ENS), qijin1@weber.edu
- Jamie Stein, instructor in ENS, jamiestein@weber.edu

- Sara Phlypo, ENS Lab coordinator, saraphlypo1@weber.edu

Following WSU undergraduate students will be assisting this project for undergraduate-level research experience:

- **Lindsey Johnson, undergraduate student, lindsayjohnson2@mail.weber.edu**
- **Chandler Williams, undergraduate student, chandlerwilliams@mail.weber.edu**
- **Alonna Jones, undergraduate/future graduate student, alonnajones@mail.weber.edu**
- **Baylee Gamble, undergraduate student, bayleegamble@mail.weber.edu**

Following are the recent WSU graduates of Spring 2024 who will be assisting this project:

- **Mason Masters, masonmasters@mail.weber.edu**

The following non-WSU affiliated individual will also be assisting with data collection and analysis for graduate-level research experience:

- Anthony Ludwig, graduate student at SJSU, anthony.f.ludwig@gmail.com
- **Jim Healis, former WSU undergraduate student and former SUS graduate student, no current affiliation, jhealis@gmail.com**

*required

Study Site

Please select all sites at which research will be conducted.

✓ Weber State University

At which Weber State University facilities will this research take place. (select all that apply)

Main Campus - Ogden

West Center

Farmington Station

Community Education Center - Ogden

Center for Continuing Education - Clearfield

Davis

Morgan Center

Other WSU affiliated site(s)

✓ External Site (non Weber State University)

Please provide the names of the external collaborating sites.

Some data collection will be conducted in our reserved sites near the finish line and vendors of the Ogden Marathon Expo in downtown Ogden. Blood and urine samples will be stored/analyzed in the Nutrition biochemistry lab in Swenson Hall.

Study Dates

Please provide the PROJECTED study start and end dates. Projects may not begin until approval letter is received.

*required

Start Date

01-01-2024

*required

End Date

12-31-2024

Departmental Approval

*required

Does your Department require approval of IRB submissions?

✓ Yes

*required

Please upload a signed departmental approval form.

Download Form

Departmental Form IRB Ogden Marathon.pdf

No

4- Subject Information

Subject Enrollment

Enter the number of subjects that will be enrolled in this study.

*required

Total Study Enrollment

Please enter the total number of subjects to be enrolled at all study sites.

50. We are enrolling 20 subjects for the full protocol. We would like to recruit 30 more subjects for a partial protocol (these subjects would only complete surveys/questionnaires).

*required

Enrollment at Weber State University

*Please enter the number of subjects that will be enrolled at **Weber State University**.*

0-50

*required

Ages

Select the age range of subjects that will be enrolled in this study. Check all that apply.

Fetus

Birth to less than 1 month

1 month to less than 12 years old

12 years old and less than 18 years old

✓ 18 years and older

*required

Vulnerable Populations

*Please check the population(s) that will be **TARGETED** for enrolled. Check all that apply.*

Fetuses

Pregnant Women

Minors with Parental Consent

Minors who can Consent Themselves (emancipated minors, minors in states that allow consent; please direct any questions about this to the IRB office)

Prisoners

Individuals with Cognitive Impairments

Economically Disadvantaged Persons

Educationally Disadvantaged Persons

Other

✓ None of the Above

5- Study Design

*required

Research Summary and Justification

Please provide a detailed summary that includes background and justification for the proposed research.

Please limit this section to 1000 words, citations should be included as a separate Appendix if necessary.

This research proposal is inherently an extension of our department's (Exercise and Nutrition Sciences; ENS) recently awarded Ambrose Amos Shaw Endowed Chair Award. This was awarded to promote a collaborative research opportunity for faculty and students alike to investigate characteristics and biomarkers in marathon runners.

Marathon running induces considerable physiological, psychological, and biomechanical strain on participants (Sanchez, Corwell, & Berkoff, 2006). The duration of exercise required to complete a marathon increases biological markers of cardiac stress (e.g. Troponin T), inflammation (e.g. TNF-α), and intestinal permeability (e.g. Fatty Acid Binding Protein) (Bernat-Adell et al., 2019; Da Ponte et al., 2018; Karhu et al., 2017; Scherr et al., 2011). Further, nutritional practices leading up to and during a marathon have considerable impacts on performance and recovery (Almond et al., 2005; Mielgo-Ayuso et al., 2020). The repetitive eccentric contractions occurring during running combined with the long distance (26.2 miles), provides the perfect scenario to induce considerable muscle damage (Clarkson, 2007; Del Coso et al., 2013; Kyröläinen et al., 2000). As such, many biological and biomechanical markers representing this damage are elevated following a marathon (Del Coso et al., 2013; Mielgo-Ayuso et al., 2020). This may have impacts on many biomechanical properties that affect gait, stride length, stride rate, running economy, and ultimately function (Del Coso et al., 2013; Kyröläinen et al., 2000). Muscle damage not only leads to biomechanical changes and soreness, but also challenges other physiological systems to maintain function. For example, certain cellular contents released from damaged muscle fibers (e.g. myoglobin) into the circulation can have nephrotoxic effects (i.e. damage the kidneys), which may compromise overall renal function (Bosch, Poch, & Grau, 2009; Clarkson, 2007). When running a marathon, the renal system is stressed due to reduced perfusion associated with exercise and is often combined with hypovolemia from dehydration (Mansour et al., 2017; Sanchez et al., 2006). Therefore, adding a toxic substance such as myoglobin, damages the nephrons and leads to elevations in biological markers of acute kidney injury post-race (e.g. creatinine, kidney injury molecule-1) (Mansour et al., 2017; McCullough et al., 2011; Mingels, Jacobs, Kleijnen, Wodzig, & Dieijen-Visser, 2009). The responses to marathon running are variable in the literature, due to differences in weather, altitude, and course design. This variation is beneficial for providing a wide response pattern and allows for a personalized assessment for expected results given specific conditions.

As such, the purpose of this study is to evaluate nutritional behaviors and the biomechanical and physiological responses to running the Ogden Marathon. This marathon course contains a considerable downhill component, which facilitates muscle damage more-so than flat marathons. Therefore, we expect considerable physiological and biomechanical strain that may lead to delayed recovery. Further, we intend to assess variables pre-marathon, immediately post-marathon, and at 48 hours post-marathon. Unfortunately, relatively few studies have evaluated the effects of completing a marathon past 24 hours, likely due to participant availability. The relative lack of data for responses past 24 hours is concerning as

many biological markers do not peak until as late as 72 hours (e.g. creatine kinase) or may have variations that peak immediately and then again after several days (e.g. kidney injury molecule 1). Thus, we propose to investigate these variables at various time points to contribute to a needed area in research.

Appendix

Please attach appendix document(s) as needed.
Appendix A - References.docx

*required
Hypothesis

Provide the study hypothesis.
We hypothesize that variables associated with muscle damage will remain elevated at the 48-hour follow-up, however only certain physiological variables (e.g. kidney injury molecule-1) will be increased following recovery.

*required
Objectives

Provide the study objectives.
The Ogden Marathon is unique in that its course has a considerable downhill component. This uniqueness allows us to analyze and contribute to the growing body of variable response patterns for various marathons.

- Assess baseline biomarkers as well as up to 48 hours post-marathon for analysis.
- Analyze health patterns, biomechanics, and nutrition habits to further explore potential covariates and relationships.

*required
Outcome Measures

Provide the main study outcome measures/dependent variables.

We will mainly be assessing muscle damage biomarkers and hydration markers via blood and urine samples at various time points. We will also be collecting the following outcome measures to assess further relationships:

- Scales for thirst perception (detailing the level or sensation of thirst), muscle pain (how much pain they are feeling), and fatigue (how tired they feel) to evaluate the perceptual strain.
- Body weight and composition before and after the race
- Skin carotenoid levels before and after race
- Training/medical history
- Questionnaires detailing participant NSAID use will be completed before, following, and after 48 hours of recovery.
- Dietary patterns such as caloric intake and nutrient breakdown
- Run/stride frequency during race
- Isometric strength before and after race

*required

Inclusion Criteria

List and describe the inclusion criteria.

- Participants to be between the ages of 18–65 years old
- Participants are already registered and running the full Ogden Marathon

*required

Exclusion Criteria

List and describe the exclusion criteria.

Exclusion Criteria is only for the full protocol:

- No known cardiovascular, respiratory, gastrointestinal bleeding, inflammatory, metabolic/ fluid-electrolyte disorder or other chronic diseases, or pregnant
- Prone to vasovagal syncope as a result of venipuncture
- Not able to attend all visits

6- Study Procedures

*required

Describe all study procedures.

Provide a complete and thorough description of the procedures in the proposed research.

This description should encompass the experimental course of a subject from their entry into the study to their completion of the study.

This study's protocol is multi-faceted. We will recruit subjects through Ogden's GOAL (Get Out And Live) Foundation whom we have already been approved and partnered with for race day logistics (see attached MOU in the Appendix). This study will consist of a familiarization and three visits (pre-race expo, race day, and 48-hour follow-up). The familiarization (in-person or virtual Zoom) will include completing the informed consent form, followed by dietary surveys (Appendix) and a 24 h history questionnaire (Appendix) The participant will also be enrolled in a Strava group with de-identified codes. Following completion of thes forms, demographic information (e.g. height, weight, age, gender, and ethnicity) will be collected. Meeting times will then be scheduled for the day before the race (at the expo) and the morning of the race.

During visit one at the expo, participants will also be instructed to record dietary intake for prior to the race, during the race day, and following the race using the automated self-administered 24 hr recall (ASA24-202 Appendix). The participants will be provided a login specific to their participant ID, but deidentified to protec their identity. Participants will also complete a training/health history survey and body weight/composition assessment. Lastly, they will be given a urine sample collection kit and further instructions, checks, and reminders for race day.

On the day of the race (visit two), the participant will report to the designated area near the shuttle location that will transport them to the race start line. Upon arrival, participants will be asked to provide the urine sample in a urine specimen cup and have their body mass measured on a standard research body mass scale. Hydration will be analyzed using urine specific gravity via refractometer, urine color, and urine osmolality via freezing point depression (Advanced Instruments osmometer). Urine will also be stored for la analysis of biological markers (i.e. inflammatory markers, renal biomarkers, cytokines, immune markers, markers of metabolism, cardiovascular markers). Participants will place their hand on our carotenoid senso to measure skin carotenoid levels. Participants will also complete a pre-race questionnaire (Appendix), pair fatigue, and thirst scales (Appendix) as well as record body weight/body composition through our InBody scale. They will also be tested for isometric leg strength on both legs with a portable force transducer. A sm subset of runners (n=10) will also be instrumented with a RunScribe device on their shoe that will track biomechanical information. A small blood sample (~20 mL or ~1.4 tablespoon/one tube) will be obtained via venipuncture from the antecubital vein from trained phlebotomists. Blood samples will be used to analyze osmolality and saved for later analysis of biological markers (i.e. inflammatory markers, renal biomarkers, cytokines, immune markers, markers of metabolism, cardiovascular markers). Participants will then be provided a wristband or stamp that will indicate their participation for identification after the race by researchers. They will then go complete the race.

Upon finishing the race, the participants will be asked to report to the data collection tent near the finish line Upon reporting the participant will provide a urine sample, body mass/composition measurement, and bloo sample via venipuncture from the antecubital vein (~20 ml or one tube). Participants will also complete a post-race questionnaire (Appendix), pain, fatigue, and thirst scales, and provide a description of their dietar intake during the race. They will also be re-tested for isometric leg strength on both legs. The runners wear

the RunScribe units will also be de-instrumented. Participants will then be provided beverages and snacks desired) and they will schedule a time to come to their 48-hour follow-up visit at the Weber State University Human Performance Laboratory. Participants will continue to record dietary intake using the ASA24-2022 f an additional 2-days post-race.

Upon arriving for the 48-hour follow-up visit (visit three), participants will provide a urine sample, body mass measurement, and blood sample via venipuncture from the antecubital vein (~20 ml or two tubes). Participants will also complete a recovery questionnaire (Appendix J), pain, fatigue, and thirst scales. They will also be tested for isometric leg strength on both legs. Participants will also be instructed to change into tight fitting clothing (e.g. spandex shorts, sports bra, bathing suit) to complete a BodPod assessment of boc composition. Once complete the participants will be provided a copy of their BodPod results.

For participants who elect to partake in the partial protocol, they will only have to complete the familiarizatic and complete the online ASA24 dietary recalls on the same days as the full protocol.

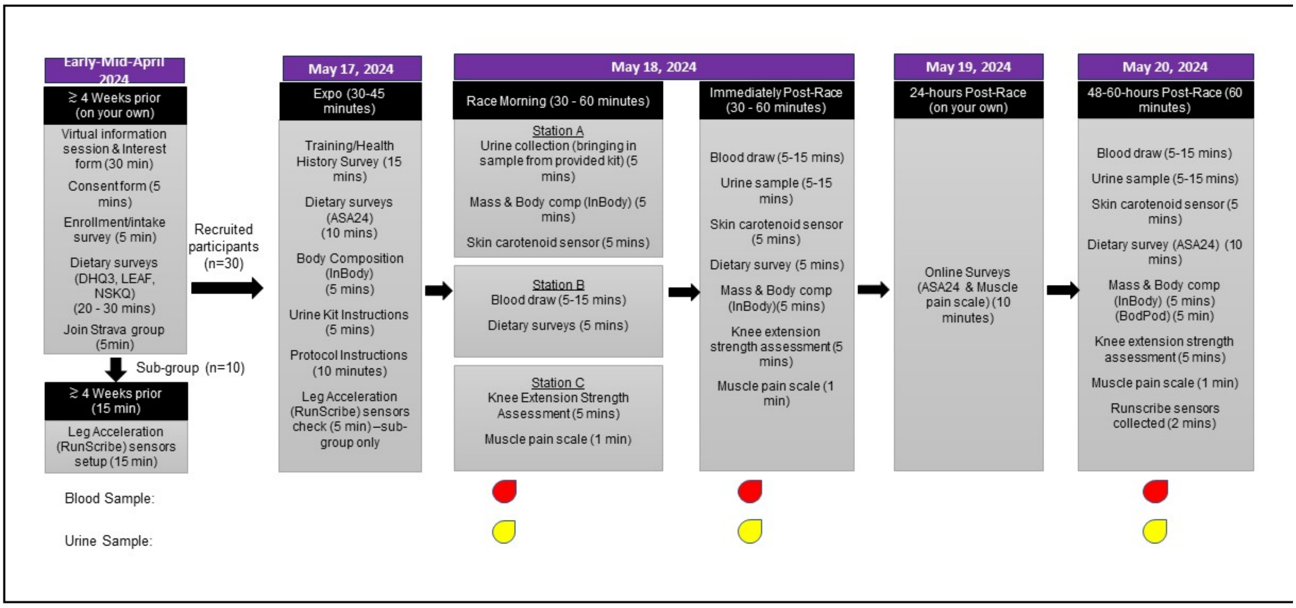

*required

Describe your subject recruitment procedures and any material inducements given for participation

Recruitment will occur via word of mouth, emails sent to race participants, and social media postings such as Facebook pages. Recruitment statements will read as follows: Participants needed for study runners completing Ogden Marathon.

- Participants (up to 20) for the full research protocol including recording your diet and providing urine and blood samples can receive a VIP shuttle to the marathon starting line, report of body composition via BodPod, and $100 gift card after completing the study.
- Additional participants are sought to completes surveys only (no urine and blood samples). The survey-only participants are not eligible for the gift card or a body composition analysis.

In order to participate, you must be already registered for and run the Ogden Full Marathon and not pregnant. If interested, please visit to this link (link to an online interest form). If you have any questions, contact Dr. Saori Hanaki at saorihanaki@weber.edu, Dr. Stacie Wing-Gaia at swinggaia@weber.edu, Dr. Bryan Dowdell at bryandowdell@weber.edu, Dr. Damon Joyner at damonjoyner@weber.edu, Dr. Qi Jin at qijin1@weber.edu, or Dr. David Aguilar Alvarez at daguilaralvarez@weber.edu.

*required

Describe the duration of study participation, the length and number of study visits, and the timetable for study completion.

Please be sure to provide the following:

- How many times human subjects will perform research activities;
- How long each visit will take;
- Total time requirement for a subject to complete the proposed research?

Participants will complete a familiarization visit and three additional visits (pre-race expo, race day, and 48-hour recovery day). The familiarization should take ~30 minutes. The pre, post, and 48 hour recovery visits will take ~30 minutes. The surveys completed on their own take ~15 minutes each. Therefore total time commitment is ~3 hours.

*required

Describe the information to be gathered and the means for collecting and recording data.

Many studies use multiple instruments/questionnaires/surveys as part of the research methodology. If applicable to the proposed research, list each instrument/questionnaire/survey that will be administered to subjects and provide a rationale for the inclusion of each one.

If previously collected data is to be used, describe both the previous and proposed uses of these data.

- Hydration will be assessed via a refractometer, urine color chart, and osmometer from urine samples.
- Blood biomarkers associated with muscle damage will be assessed via assay kits.
- Scales for thirst perception (detailing the level or sensation of thirst), muscle pain (how much pain they are feeling), and fatigue (how tired they feel) to evaluate the perceptual strain.
- Body weight and composition via Inbody scale and Bod Pod.
- Skin carotenoid levels via skin carotenoid sensor.
- A training/medical history questionnaire will be used to evaluate health history and quantify the level of activity in which individuals regularly engage.
- Questionnaires detailing participant NSAID use will be completed before, following, and after 48 hours of recovery.
- A validated online diet recording survey (ASA24) as well as DHQ3, LEAF, NSKQ will be used to quantify macronutrient and micronutrient intake for the duration of the study.
- RunScribe sensors will be attached to select participants' shoes to identify their biomechanical responses.
- Isometric strength will be assessed using a force transducer to identify changes associated with muscle damage.

Study Instruments

Attach all instruments (i.e. personality scales, questionnaires, evaluation blanks, etc) to be used in the study.

ACSM Health History & Physical Activity Questionnaire.pdf

Nutrition Knowledge Survey.docx

McKay Training Status Classification.pdf

The LEAF-Questionnaire.pdf

Sample questionnaire DHQ3.pdf

ASA24 Info.docx

Ogden Marathon Data Collection Checklist.docx

*required

Survey, Questionnaire, or Interview

Will the study utilize surveys, questionnaires, or interviews?

✓ Yes

*required

Attach all copies of surveys, questionnaires, or interviews.

If your survery is hosted on an online application, WSU IRB prefers a link to the study. Please use the attached link function.

ACSM Health History & Physical Activity Questionnaire.pdf

Nutrition Knowledge Survey.docx

McKay Training Status Classification.pdf

The LEAF-Questionnaire.pdf

Sample questionnaire DHQ3.pdf

ASA24 Info.docx

Ogden Marathon Data Collection Checklist.docx

No

*required

Will the survey, questionnaire, or interview record any information that can identify the participants?

Yes

✓ No

*required

Genetic Testing

Will this study involve genetic testing?

Yes

✓ No

*required

Drugs, Devices, Biologics

Will the study involve administering any of the following? Check all that apply.

Drug

Biologic

✓ Device

*required

Please describe.

A RunScribe motion sensor device will be placed on a subset of the subject's shoes during the race.

None of the above

*required

Participant Data, Specimens, and Records

Does this project involve the collection or use of materials (data or specimens) recorded in a manner that could identify the individuals who provided the materials, either directly or through identifiers linked to these individuals?

Yes

✓ No

*required

Specimen and Sample Retention

Does the research team plan to retain any Specimens or Biologic samples for future analysis.

✓ Yes

*required

Please explain the intent of the retention and expected destruction date.

NOTE: Intend to retain samples for future analysis **MUST** be clearly stated in your informed consent document and disclosed to participants.

All blood and urine samples will be properly stored in our biochemistry lab (SW 133) for future biomarker analysis. These samples will be retained for the maximal allowable time of three years after which all samples will be properly disposed of according to university and OSHA protocols.

No

7- Participant Protection

***Risk:** Generally, risk assessment in research considers the harm, trauma, discomfort, stress, or any other undesirable or untoward consequence of being a research subject whether anticipated or unexpected. Risk may take the following forms: physical, psychological, emotional, financial, and/or social. This list is representative but not comprehensive.*

Please consider the following questions based on the experiences that subjects might encounter through participation in the proposed research.

---

*required

Potential Risk

Whether great or small, do you think there is potential risks to subjects that participate in the proposed research.

✓ Yes

No

Potential Risks

---

*required

Describe immediate risks, long-term risks, rationale for the necessity of such risks, alternatives that were or will be considered, and why alternatives may not be feasible.

There are multiple risks associated with this study. Most of these risks are inherent and cannot be avoided because of the purpose of the study, thus alternatives are not feasible.

The full protocol (includes blood/urine samples) risks are:

- Inherent risks associated with voluntary exercise and exertion
- Inherent risks associated with blood draws such as feelings of discomfort and pain associated with needles as well as vasovagal syncope (fainting upon sight of blood/needles)

- Subjects are required to answer questions from health surveys or questionnaires such as dietary recalls that may induce feelings of discomfort.
- We cannot guarantee absolute confidentiality

The partial protocol (only includes surveys/questionnaires) risks are:

- Subjects are required to answer questions from health surveys or questionnaires such as dietary recalls that may induce feelings of discomfort.
- We cannot guarantee absolute confidentiality

What steps will be taken by the investigator to reduce the aforementioned risks?

- Exclusion criteria of pre-existing conditions to mitigate inherent risks associated with exercise (anybody with CVD diseases, pulmonary conditions, etc cannot participate).
- Exclusion criteria of those weary of blood draws (i.e. prone to vasovagal responses such as fainting upon the sight of blood/needles during blood draws).
- We will be using trained phlebotomists for the blood draws. Universal precautions (PPE) will be utilized to minimize any chance of infection. The participants will be in a seated position during the blood draw (minimizing any injury should they feel lightheaded). Also, pressure will be applied to the blood draw sight to minimize soreness.
- Subjects will be reminded that this study is entirely voluntary. If they want to discontinue participating, they may drop out at any time and contact research personnel.
- All surveys and assessments will be completed by a trained research staff member.
- All data will be de-identified.

*required

Describe any potential legal, financial, social, or personal affects on subjects.

None

What steps will be taken by the investigator to reduce the aforementioned risks?

---

*required

Will deception be used as a method of data gathering?

Yes

✓ No

*required

Does this research require face to face contact between researchers and participants

---

✓ Yes

*required

Please detail your COVID 19 Mitigation Procedures.

If required by the university, the IRB, and/or the state of Utah, all subjects and researchers will wear masks (face coverings) at all times.

No

*required

Expected Benefits

Please describe any benefits that research subjects will receive as a direct result of their participation in the proposed research.

Note: *compensation is not considered a benefit*

Subjects will be given interesting information about their heath habits and body composition through assessments that normally cost money. In addition, subjects are given access to complimentary and private access to a bus shuttle and restrooms before and after the marathon designated only for study subjects.

Please describe how this research may provide benefit to scientific knowledge, a specific discipline, and/or society in general.

By participating and contributing to data collection, subjects can know that the findings from this research will contribute to a missing area of research in terms of marathon responses.

*required

Subject Compensation

*required

Will subjects receive any form of compensation for participation in this investigation?

Compensation includes but is not limited to: University Credit, extra points in a course, gift cards or any form of monetary instrument.

✓ Yes

No

*required

Please describe and detail any and all forms of compensation that subjects may receive.

$100 gift card

*required

Safeguarding Subjects' Identity

What uses will be made of identifiable information obtained from the subjects?

The only identifiable information (name) will be the signed informed consent form. Signed informed consent forms will be kept separated from data collected, and the signed/completed hard copy documents will be stored in a locked cabinet in our (ENS) lab coordinator's office (Sara Phlypo who is part of research personnel).

What precautions will be taken to safeguard identifiable records or individuals?

The only identifiable information (name) will be the signed informed consent form. Signed informed consent forms will be kept separated from data collected, and the signed/completed hard copy documents will be stored in a locked cabinet in our (ENS) lab coordinator's office (Sara Phlypo who is part of research personnel).

*required

Data Management

Protecting ***hard copy*** data may involve de-identification of data (see:NISTIR-8053) and secured storage locations and conditions (see: Weber State University PPM 10-1).

Describe what type of hard copy data will be generated by the proposed research (i.e.,

notes, audio/video tapes, questionnaires, etc.). Where will this hard copy data be stored and how will it be protected?

The only identifiable information (name) will be the signed informed consent form. Signed informed consent forms will be kept separated from data collected, and the signed/completed hard copy documents will be stored in a locked cabinet in our (ENS) lab coordinator's office (Sara Phlypo who is part of research personnel).

Hard copy surveys/questionnaires (such as the nutritional and dietary surveys) and the stored biologicals samples (in SW 133 biochemistry lab) will use de-identified codes (i.e. OMS-01). Hard copy data will be stored securely and separately from the informed consents in our lab coordinator's office. Biological samples will be stored securely in our -80 degree freezer in SW 133 biochemistry lab in which only authorized users have access.

Protecting ***electronic data*** may involve a secure network, password access, and data de-identification/ encryption (see: Weber State University PPM 10-1)

Describe what type of electronic data will be generated by the proposed research (i.e., computer files/ spreadsheets, questionnaires, images, video, audio/mp3 files, etc.) Where will this electronic data be stored and how will it be protected

All questionnaire results (de-identified) will be entered into data spreadsheets saved in a Box/Google Drive Folder which only the study investigators will have access to. These are password-protected and managed by the university. The ASA24 platform data are stored under a subject's ID number without any identifiable information. ASA24 platforms are password-protected and only the research personnel will have access to it. The exported data from this platform will be stored in the Google Drive/Box mentioned above.

*required

Will the research require accessing student educational records?

Yes

✓ No

*required

On what date will study data and materials with identifiable information be destroyed?

Please select a year and date.

12-31-2027

*required

Will raw data be made available to anyone other than the principal investigator and the immediate study personnel?

Yes

✓ No

*required

Informed Consent

Describe the procedures for obtaining informed consent.

Informed Consent will be obtained in either hard-copy form or virtually via a live Zoom session with recruited participants. We will have two separate Informed Consent forms: one for those interested in completing the full protocol (which includes blood draws and urine samples) and one for the partial protocol (which only includes the survey/questionnaires).

*required

Please attach the your Informed Consent Document(s). Please include all versions/languages you intend to utilize. **PDF format required!**

You will also have a chance to attach on the last page of the application.
Download Template (Word Doc Format)
Informed Consent Full Protocol.doc

Informed Consent Partial Protocol.docx

8- Conflict of Interest

*required

Do you or any investigator(s) participating in this study have a financial interest related to this research project?

Yes

✓ No

Study Funding

*required

Does this study have **EXTERNAL** funding

Yes

✓ No

*required

Does this study have an **INTERNAL** funding sources

✓ Yes

Please list sources of INTERNAL funding.

Ambrose Amos Shaw Endowed Chair Award

No

9- Attachments

Participant Protection

Informed Consent Form

Please upload **ALL** versions of the Informed Consent document that maybe used. If requesting a waiver please upload justification here. **PDF format required!**
Informed Consent Full Protocol.doc

Informed Consent Partial Protocol.docx

If requesting an Informed Consent Waiver/Alternation under 45 CFR 46.16 please explain.

*required

Research Human Subjects Training Certificate(s)

Please upload a CITI certificate for **EVERY** investigator listed on the application
Citi Certificate - Jamie Stein.pdf

CITI certificate Damon Joyner.pdf

CITI DavidAguilar.pdf

CITI_qijin.pdf

citiCompletionCertificate_Dowdell_2023.pdf

Stacie Wing-Gaia CITI Completion certificate.pdf

citiCompletionReportHanakiJan2021.pdf

citiCompletionCertificate_Sara Phlypo.pdf

citiCompletionCertificate_Lindsay_Johnson.pdf

citiCompletionCertificate_Mason_Masters.pdf

CITI Program Completion Certificate Chandler_Williams.pdf

citiCompletionCertificate_Jim_Healis.pdf

CITI completion certificate Alonna Jones.pdf

gamblebaylee_2072570_116349305_citiCompletionCertificate_14011567_67119071 2.pdf

Study Procedures

Study Documents

If applicable, this includes flyers used for recruitment.
Recruitment announcement.docx

Study Instruments

Attach all instruments (i.e. personality scales, questionnaires, evaluation blanks, etc) to be used in the study.
ACSM Health History & Physical Activity Questionnaire.pdf

Nutrition Knowledge Survey.docx

McKay Training Status Classification.pdf

The LEAF-Questionnaire.pdf

Sample questionnaire DHQ3.pdf

ASA24 Info.docx

Ogden Marathon Data Collection Checklist.docx

FDA Letter

Study Design

Appendix
Appendix A - References.docx

External IRB of Record

Study Protocol

Attach the protocol for this study that was reviewed by the Outside IRB.

Outside IRB Approval

Attach the IRB Approval from the Outside IRB.

Outside IRB Review Meeting Minutes

Attach the minutes from the outside IRB meeting(s) for the review of this study.

Outside IRB Correspondence

Attach all correspondence concerning the review of this study by the Outside IRB.
